# Supplementary material for: Feedback control of organ size precision is mediated by BMP2-regulated apoptosis in the Drosophila eye
Source: PLoS Biol. 2024 Jan 30;22(1):e3002450. doi: 10.1371/journal.pbio.3002450 (PMC10826937; doi:10.1371/journal.pbio.3002450)

Suppl. Fig. 1 to Figure 1. Expression of the apoptosis markers activated caspase-3 (Cas3*) and activated Dcp-1 in control eye discs. (a,a') *optix>GFP* primordium stained for GFP, the photoreceptor marker Elav and Cas3*. The expression of the *optix-GAL4* driver, as detected by GFP-expression is outlined (in a'). The Cas3* signal is low (a'). (b,b') *optix>+* primordium stained for the G2-marker Cyclin B (CycB) and Dcp-1. Dcp-1 signal is detected in a band of cells anterior to the differentiating wavefront (this latter lacks CycB expression).

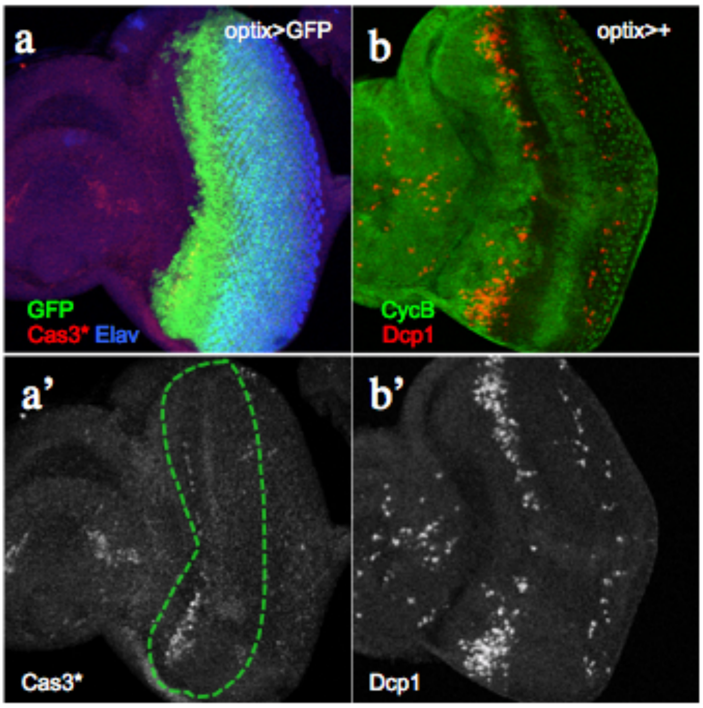

Supplement: S1 Fig — (a, a’) optix>GFP primordium stained for GFP, the photoreceptor marker Elav and Cas3*. The expression of the optix-GAL4 driver, as detected by GFP-expression is outlined (in a’). The Cas3* signal is low (a’). (b, b’) optix>+ primordium stained for the G2-marker Cyclin B (CycB) and Dcp-1. Dcp-1 signal is detected in a band of cells anterior to the differentiating wavefront (this latter lacks CycB expression). (PDF) [file pbio.3002450.s001.pdf]
