# Supplementary material for: Feedback control of organ size precision is mediated by BMP2-regulated apoptosis in the Drosophila eye
Source: PLoS Biol. 2024 Jan 30;22(1):e3002450. doi: 10.1371/journal.pbio.3002450 (PMC10826937; doi:10.1371/journal.pbio.3002450)
Supplement: S2 Fig — The average ommatidial size was calculated as the area occupied by 10 adjacent ommatidia in the equatorial region of the eye divided by 10. Independent eyes were measured on photographs of mounted eyes of the indicated genotypes. The statistical comparison of the values obtained relative to the control (“optix>+”) did not detect significant differences among genotypes. The data underlying the graphs shown in the figure can be found in “S2_Fig 1_data” in the Supporting information file S1 Raw Data. (PDF) [file pbio.3002450.s002.pdf]

Suppl. Fig. 2 to Figure 1. The average ommatidial size is approximately constant in different genotypes tested. The average ommatidial size was calculated as the area occupied by 10 adjacent ommatidia in the equatorial region of the eye divided by 10. Independent eyes were measured on photographs of mounted eyes of the indicated genotypes. The statistical comparison of the values obtained relative to the control *("optix>+")* did not detect significant differences among genotypes.

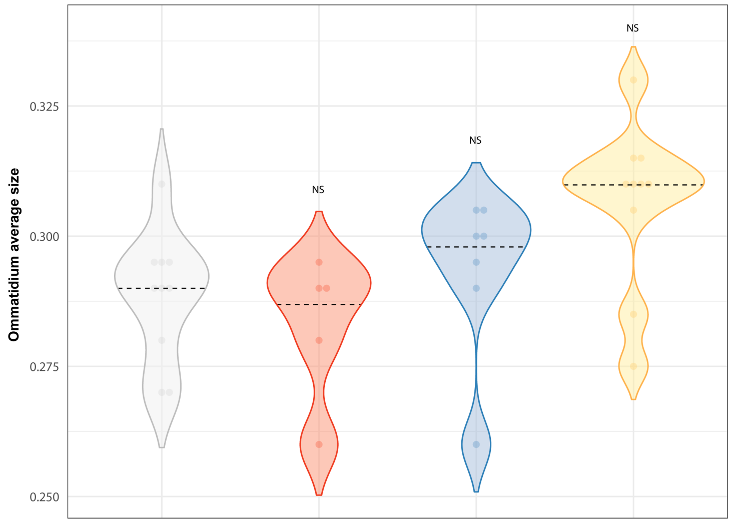

Median regression model	optix>+	optix>RHGRI	optix>tkvRI	optix>tkvRI+RHGRI
Effects	0.29	0.00	0.01	0.01
p.value	0	1	0.2187	0.5596
Predicted for group	0.29	0.29	0.30	0.31
