# Supplementary material for: Feedback control of organ size precision is mediated by BMP2-regulated apoptosis in the Drosophila eye
Source: PLoS Biol. 2024 Jan 30;22(1):e3002450. doi: 10.1371/journal.pbio.3002450 (PMC10826937; doi:10.1371/journal.pbio.3002450)
Supplement: S3 Fig — Results are shown for rE and sFAi of eyes from individuals of the following genotypes: “+”: Oregon-R (Or-R) wild-type strain; “optix>+”: Progeny from the cross optix-GAL4 to Or-R; “optix>UAS-CherryRI”: The optix-GAL4; UAS-Cherry_RNAi progeny obtained by crossing optix-GAL4 to UAS-Cherry_RNAi. This dataset was obtained independently of the datasets included in Fig 2 of the main text. The median rE of optix>CherryRI eyes is slightly higher than that of the optix>+ reference control group; however, it is not significantly higher than that of the wild-type Or-R group (a). There are no significant differences in sFAi between any of the groups compared (b). The table shows the statistical analysis of rE and sFAi among the different groups considered. This analysis concludes that there is no significant effect of driving a nonspecific, neutral RNAi on eye size precision and that the effect on final eye size is within the variability present among the alternative control lines used (An equivalent table is explained in S2 Table. Further details on the statistical analysis can be found in the main text Materials and methods section). The data underlying the graphs shown in the figure can be found in “S3_Fig 1_data” in the Supporting information file S1 Raw Data. (PDF) [file pbio.3002450.s003.pdf]

Suppl. Fig. 3 to Figure 1. Study of the effect of a neutral RNAi on rE and sFAi values. Results are shown for rE and sFAi of eyes from individuals of the following genotypes: “+”: Oregon-R (Or-R) wild type strain; “*optix>+*”: Progeny from the cross *optix-GAL4* to Or-R; “*optix>UAS-CherryRI*”: The *optix-GAL4; UAS-Cherry_RNAi* progeny obtained by crossing *optix-GAL4* to *UAS-Cherry_RNAi.* This dataset was obtained independently of the datasets included in Figure 2 of the main text. The median rE of *optix>CherryRI* eyes is slightly higher than that of the *optix>+* reference control group, however, it is not significantly higher than that of the wild-type *Or-R* group (a). There are no significant differences in sFAi between any of the groups compared (b). The table shows the statistical analysis of rE and sFAi among the different groups considered. This analysis concludes that there is no significant effect of driving a non-specific, neutral RNAi on eye size precision and that the effect on final eye size is within the variability present among the alternative control lines used (An equivalent table is explained in Suppl. table 1 to Figure 2. Further details on the statistical analysis can be found in the main text Materials and Methods section).

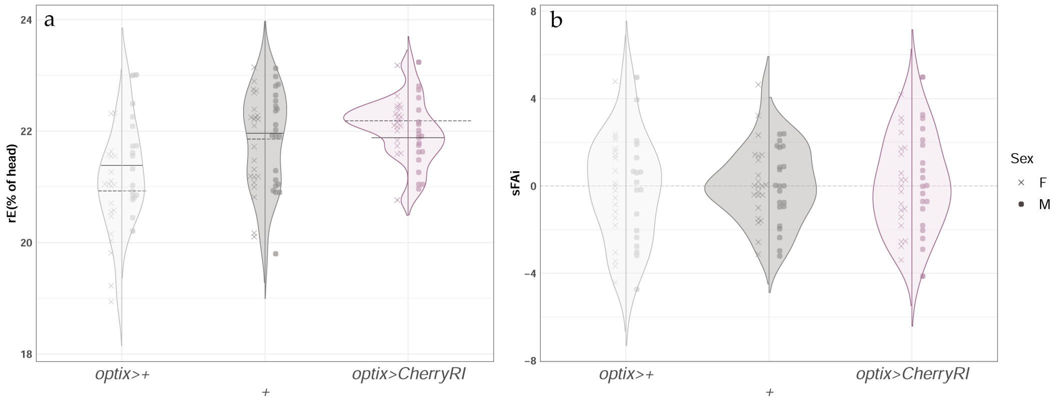

c

	rE						sFAi		
	Median effects			Dispersion analysis			Dispersion analysis		
	Effects	p.value	Predicted by group (%)	sd(j)	sd(j)/sd(optix>+)	padj.(Levene test against optix>+)	sd(j)	sd(j)/sd(optix>+)	padj.(Levene test against optix>+)
optix>+	21.05	0.0000	21.05	0.86	1.00	NA	2.41	1.00	NA
+	0.97	0.0009	22.02	0.86	1.00	0.7202	1.74	0.72	0.0722
optix>CherryRI	1.05	0.0000	22.09	0.54	0.63	0.0151	2.19	0.91	0.6170
