# Supplementary material for: Feedback control of organ size precision is mediated by BMP2-regulated apoptosis in the Drosophila eye
Source: PLoS Biol. 2024 Jan 30;22(1):e3002450. doi: 10.1371/journal.pbio.3002450 (PMC10826937; doi:10.1371/journal.pbio.3002450)

Suppl. Fig. 4 to Figure 1. Phenotypic rescue of genotypes with two UAS constructs cannot be explained by GAL4 "dilution effect". We compared the distributions and median values of rE (a) and sFAi (b) from *optix>tkvRI* (one UAS transgene: UAS-*tkvRI*) and *optix>tkvRI* + GFP (two UAS transgenes: UAS-*tkvRI and* UAS-GFP). In the case of eye size (a), if the presence of two UAS sequences had titrated the GAL4 molecules (effectively halving the number of GAL4 molecules per UAS sequence), the expectation would be a weakening of the phenotype (derived from a weaker expression of *tkv-RNAi*). In the case of sFAi (b) titration of GAL4 would result in a reduction of the asymmetry. However, these expectations were not observed. We used the Bayes Factor (BF; shown in plot) as a measure of the strength with which a null hypothesis is accepted or rejected. Values close to 0 allow accepting the null hypothesis: "there is no GAL-4 dilution effect". Black bars show the minimum detectable differences in each comparison depending on sample sizes and distribution shapes with 80% power (See Supplementary Statistical Methods). Indeed, we find very low BF values for both rE and sFAi, supporting the idea that there is no dilution effect of GAL4 when two UAS transgenes, instead of one, are present in the genotype. Therefore, the phenotypic effects detected are genuinely caused by the genetic perturbation.

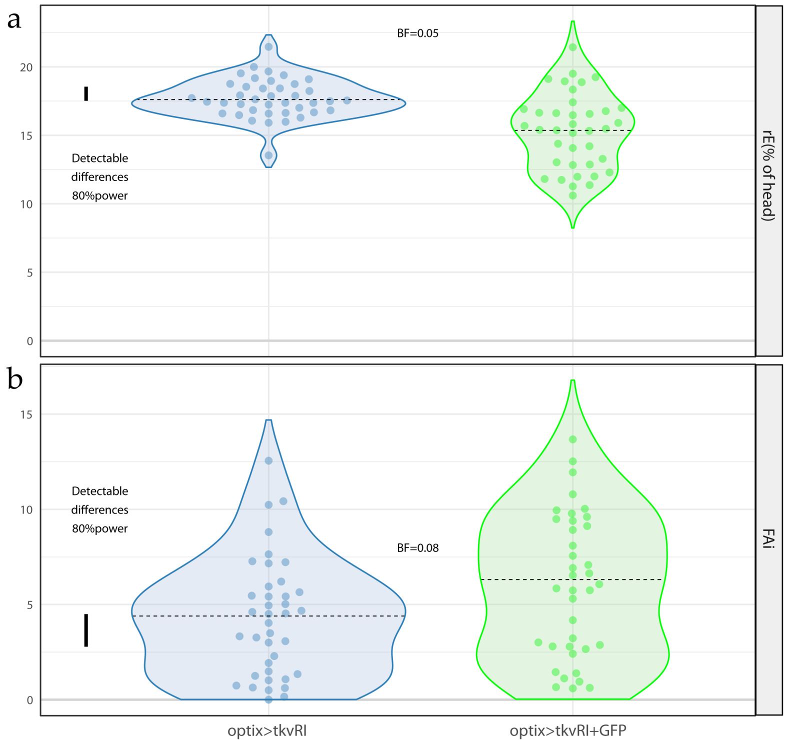

Supplement: S4 Fig — We compared the distributions and median values of rE (a) and sFAi (b) from optix>tkvRI (1 UAS transgene: UAS-tkvRI) and optix>tkvRI + GFP (2 UAS transgenes: UAS-tkvRI and UAS-GFP). In the case of eye size (a), if the presence of 2 UAS sequences had titrated the GAL4 molecules (effectively halving the number of GAL4 molecules per UAS sequence), the expectation would be a weakening of the phenotype (derived from a weaker expression of tkv-RNAi). In the case of sFAi (b), titration of GAL4 would result in a reduction of the asymmetry. However, these expectations were not observed. We used the Bayes Factor (BF; shown in plot) as a measure of the strength with which a null hypothesis is accepted or rejected. Values close to 0 allow accepting the null hypothesis: “There is no GAL-4 dilution effect.” Black bars show the minimum detectable differences in each comparison depending on sample sizes and distribution shapes with 80% power (see S1 Statistical Methods). Indeed, we find very low BF values for both rE and sFAi, supporting the idea that there is no dilution effect of GAL4 when 2 UAS transgenes, instead of one, are present in the genotype. Therefore, the phenotypic effects detected are genuinely caused by the genetic perturbation. The data used in the graphs shown in the figure can be found in “S4_Fig 1_data” in the Supporting information file S1 Raw Data. (PDF) [file pbio.3002450.s004.pdf]
