# Supplementary material for: Feedback control of organ size precision is mediated by BMP2-regulated apoptosis in the Drosophila eye
Source: PLoS Biol. 2024 Jan 30;22(1):e3002450. doi: 10.1371/journal.pbio.3002450 (PMC10826937; doi:10.1371/journal.pbio.3002450)

Suppl. Figure 5 to Figure 1. Increased cell death and fragmented *dpp* expression in *Bar* discs. L3 *dpp-Z* discs (a,b), stained for apoptosis (dcp-1, green), *dpp* transcription (beta-galactosidase, red) and the retinal differentiation marker Elav (blue). (a) control ("+": Oregon-R) and (b) *Bar* mutant discs. The ellipse approximately outlines the eye region. "*a*" marks the antennal primordium.

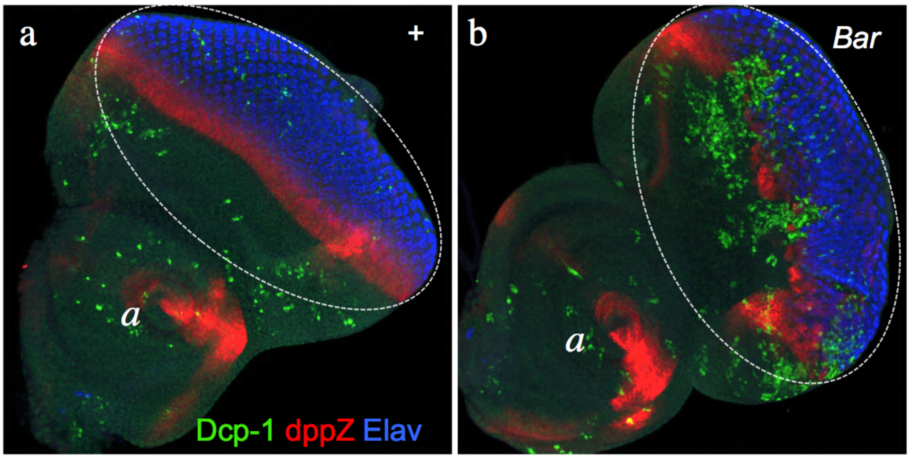

Supplement: S5 Fig — L3 dpp-Z discs (a, b), stained for apoptosis (dcp-1, green), dpp transcription (beta-galactosidase, red), and the retinal differentiation marker Elav (blue). (a) Control (“+”: Oregon-R) and (b) Bar mutant discs. The ellipse approximately outlines the eye region. “a” marks the antennal primordium. (PDF) [file pbio.3002450.s005.pdf]
