# Supplementary material for: Feedback control of organ size precision is mediated by BMP2-regulated apoptosis in the Drosophila eye
Source: PLoS Biol. 2024 Jan 30;22(1):e3002450. doi: 10.1371/journal.pbio.3002450 (PMC10826937; doi:10.1371/journal.pbio.3002450)
Supplement: S6 Fig — The intraindividual FA (FAi) is calculated using the left and right eyes from the same individual. The interindividual FA (FAn) is calculated as the difference between the left eye of an individual and the right eye randomly chosen from the population of measured eyes. The difference between FAn and FAi was computed for each fly (ΔFA). If there were no “individual” effect, ΔFA should be 0. The effect of each genotype on ΔFA median, their p.values, and the predicted median for each of them are shown (see S1 Statistical Methods). The ΔFA median is slightly, though significantly greater than 0 across all genotypes relative to the control (optix>+). This result indicates that eye size within an individual is slightly correlated irrespective of genotype. The data used in the graphs shown in the figure can be found in “Fig 1_S6Fig 1_data” in the Supporting information file S1 Raw Data. (PDF) [file pbio.3002450.s006.pdf]

Suppl. Fig. 6 to Figure 1. Analysis of intraindividual eye size correlation. The intra-individual FA (FAi) is calculated using the left and right eyes *from the same individual.* The inter-individual FA (FAn) is calculated as the difference between the left eye of an individual and the right eye randomly chosen from the population of measured eyes. The difference between FAn and FAi was computed for each fly (ΔFA). If there were no "individual" effect, ΔFA should be 0. The effect of each genotype on ΔFA median, their p.values and the predicted median for each of them are shown (See Supplementary Statistical Methods). The ΔFA median is slightly, though significantly greater than zero across all genotypes relative to the control (*optix>+*). This result indicates that eye size within an individual is slightly correlated irrespective of genotype.

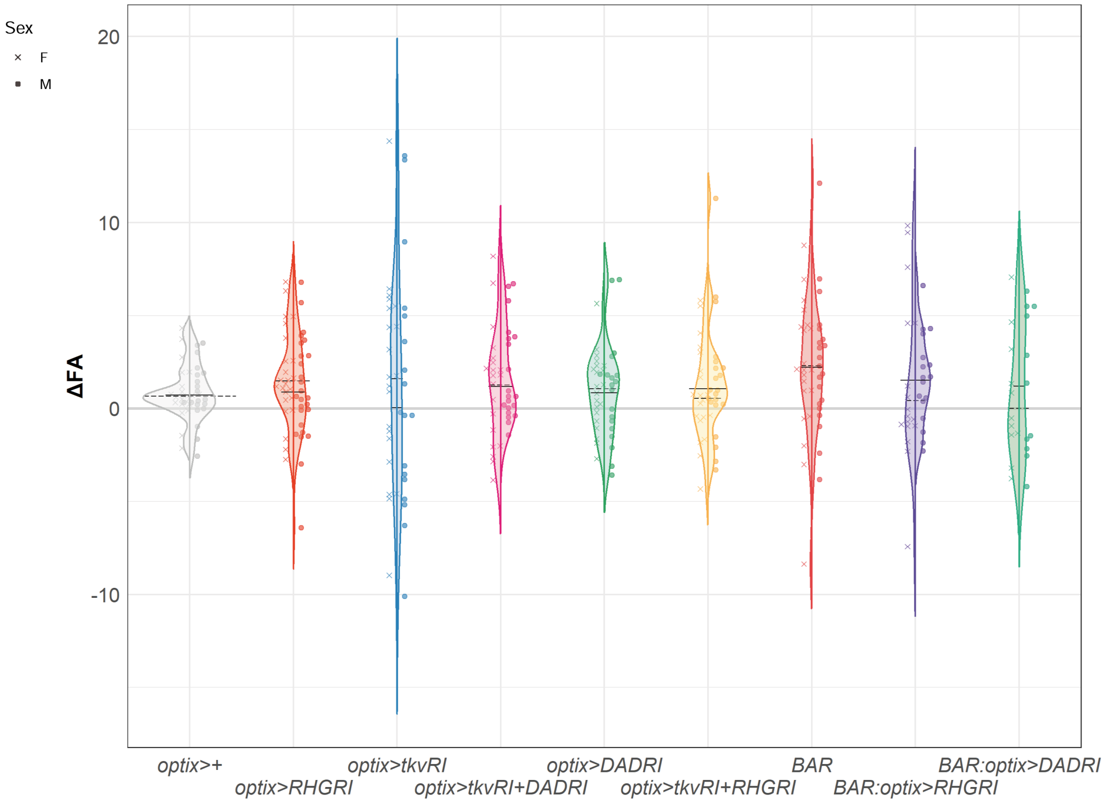

Effects	0.38	0.32	0.03	0.34	0.30	0.26	1.45	-1.15	-0.93
p.value	0.029	0.229	0.454	0.333	0.238	0.363	0.040	0.149	0.259
Predicted by group	0.38	0.71	0.41	1.34	0.68	1.00	1.84	0.72	1.21
