# Supplementary material for: Feedback control of organ size precision is mediated by BMP2-regulated apoptosis in the Drosophila eye
Source: PLoS Biol. 2024 Jan 30;22(1):e3002450. doi: 10.1371/journal.pbio.3002450 (PMC10826937; doi:10.1371/journal.pbio.3002450)

Suppl. Fig. 1 to Figure 2. Attenuation of the Dpp signaling pathway does not affect the mitotic rate of eye progenitors. (a, b) Representative late L3 eye imaginal discs, stained for the mitotic marker phosphor-histone H3 (PH3), and counterstained with rhodamine phalloidin (“actin”). (c) Distribution of mitotic rate, measured as the rate of PH3-positive area relative to the % of available area (non-apoptotic) in the anterior domain (regions outlined in yellow) in the two genotypes (See Supplementary Statistical Methods). Bayes Factor (BF) value close to 0 allows accepting the null hypothesis: “there is no significant differences between genotypes”. BF= 0.37. The bar indicates an 80% power to detect differences. This analysis indicates that mitotic rate of progenitors does not change in *optix>tkvRI* relative to *optix>+* controls (See Supplementary Statistical Methods).

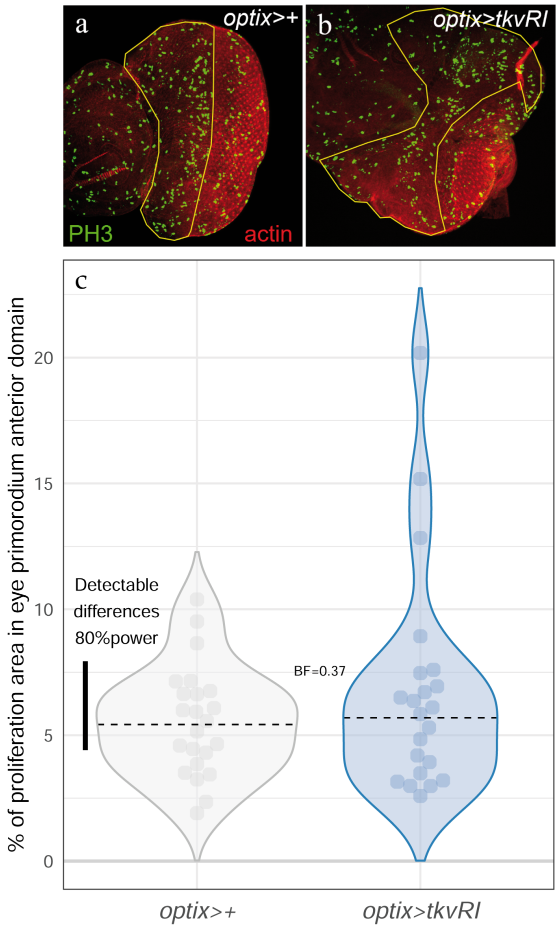

Supplement: S7 Fig — (a, b) Representative late L3 eye imaginal discs, stained for the mitotic marker phosphor-histone H3 (PH3), and counterstained with rhodamine phalloidin (“actin”). (c) Distribution of mitotic rate, measured as the rate of PH3-positive area relative to the % of available area (nonapoptotic) in the anterior domain (regions outlined in yellow) in the 2 genotypes (see S1 Statistical Methods). Bayes Factor (BF) value close to 0 allows accepting the null hypothesis: “There is no significant differences between genotypes.” BF = 0.37. The bar indicates an 80% power to detect differences. This analysis indicates that mitotic rate of progenitors does not change in optix>tkvRI relative to optix>+ controls (see S1 Statistical Methods). The data used in the graphs shown in the figure can be found in “S1_Fig 2_data_A” and “S1_Fig 2_data_B” in the Supporting information file S1 Raw Data. (PDF) [file pbio.3002450.s007.pdf]
