# Supplementary material for: Feedback control of organ size precision is mediated by BMP2-regulated apoptosis in the Drosophila eye
Source: PLoS Biol. 2024 Jan 30;22(1):e3002450. doi: 10.1371/journal.pbio.3002450 (PMC10826937; doi:10.1371/journal.pbio.3002450)

Suppl. Fig. 2 to Figure 2. Caspase 3-associated apoptosis is induced by Dad-mediated Dpp signal attenuation. *optix2/3-GAL4; UAS-Dad ("optix>Dad")* eye disc stained for activated caspase 3 ("cas3*"). Abundant cas3* signal is detected in cells anterior to the differentiation wave-front (dashed line). The disc is counterstained with the nuclear marker DAPI. (b,b') Close up of a cas3*-positive region. Cas3* signal (b': arrows) overlaps with pycnotic nuclei stained with DAPI (small, dense DAPI signal), indicating that cells enter an irreversible apoptotic process.

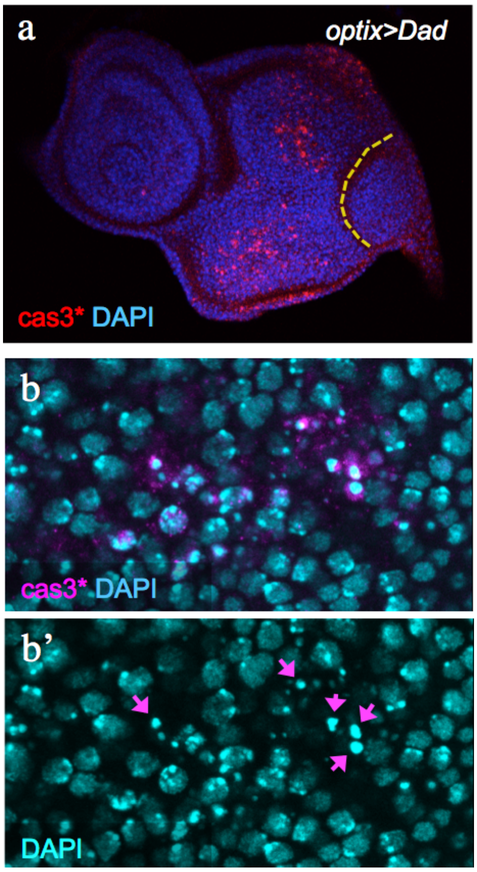

Supplement: S8 Fig — optix2/3-GAL4; UAS-Dad (“optix>Dad”) eye disc stained for activated caspase 3 (“cas3*”). Abundant cas3* signal is detected in cells anterior to the differentiation wave-front (dashed line). The disc is counterstained with the nuclear marker DAPI. (b, b’) Close-up of a cas3*-positive region. Cas3* signal (b’: arrows) overlaps with pycnotic nuclei stained with DAPI (small, dense DAPI signal), indicating that cells enter an irreversible apoptotic process. (PDF) [file pbio.3002450.s008.pdf]
