# Supplementary material for: Feedback control of organ size precision is mediated by BMP2-regulated apoptosis in the Drosophila eye
Source: PLoS Biol. 2024 Jan 30;22(1):e3002450. doi: 10.1371/journal.pbio.3002450 (PMC10826937; doi:10.1371/journal.pbio.3002450)

Suppl. Fig. 1 to Figure 4. A mutant condition in which eye size varies dramatically preserving precision. (a, b) Heads from GMR>+ (a) and GMR>Upd (b) adult males; frontal view. While the median eye size (rE: c) of *GMR>Upd* flies is approximately 1,4 times that of control (*GMR>+*) ones (statistically significant), the fluctuating asymmetry index ("precision")(FAi: d) is indistinguishable between the two genotypes. Female (F) and male (M) distributions are shown. (e, f) Eye primordia stained for the apoptotic marker Dcp-1. Apoptotic signal is observed in both *GMR>Upd* as well as in *GMR>+* control primordia.

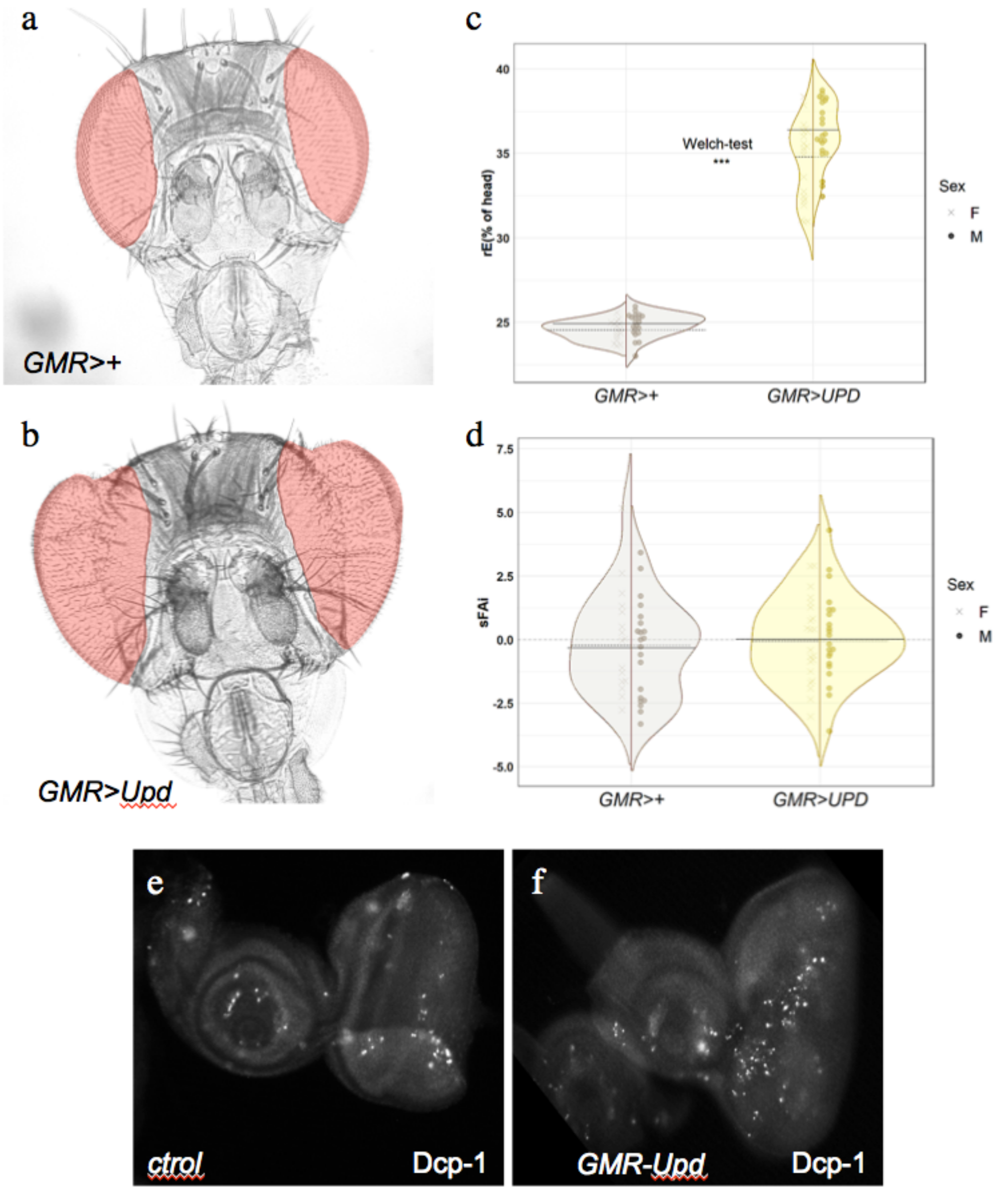

Supplement: S9 Fig — (a, b) Heads from GMR>+ (a) and GMR>Upd (b) adult males; frontal view. While the median eye size (rE: c) of GMR>Upd flies is approximately 1.4 times that of control (GMR>+) ones (statistically significant), the fluctuating asymmetry index (“precision”) (FAi: d) is indistinguishable between the 2 genotypes. Female (F) and male (M) distributions are shown. (e, f) Eye primordia stained for the apoptotic marker Dcp-1. Apoptotic signal is observed in both GMR>Upd as well as in GMR>+ control primordia. The data used in the graphs shown in the figure can be found in “S1_Fig 4_data” in the Supporting information file S1 Raw Data. (PDF) [file pbio.3002450.s009.pdf]
