# Supplementary material for: Feedback control of organ size precision is mediated by BMP2-regulated apoptosis in the Drosophila eye
Source: PLoS Biol. 2024 Jan 30;22(1):e3002450. doi: 10.1371/journal.pbio.3002450 (PMC10826937; doi:10.1371/journal.pbio.3002450)
Supplement: S1 Table — First block shows each genotype’s effect on the rE median, their p.values, and the predicted median for each of them. The second and third blocks show the standard deviation of each group for rE and sFAi, respectively, and the result of the comparison of variances of each group against the optix>+ reference group. (PDF) [file pbio.3002450.s010.pdf]

**Suppl. Table 1 to Figure 1. Analysis of the effect of genotype on rE median, rE dispersion and sFAi dispersion.** First block shows each genotype's effect on the rE median, their p.values and the predicted median for each of them. The second and third blocks show the standard deviation of each group for rE and sFAi respectively and the result of the comparison of variances of each group against the *optix>+* reference group.

| | rE | | | | | | sFAi | | |
|---|---|---|---|---|---|---|---|---|---|
| | Median effects | | | Dispersion analysis | | | Dispersion analysis | | |
| | **Effects** | **p.value** | **Predicted by group (%)** | **sd(j)** | **sd(j)/sd(optix>+)** | **padj.(Levene test against optix>+)** | **sd(j)** | **sd(j)/sd(optix>+)** | **padj.(Levene test against optix>+)** |
| *optix>+* | 21.11 | 0 | 21.11 | 0.62 | 1 | NA | 1.50 | 1 | NA |
| *optix>RHGRI* | 4.24 | 0 | 25.35 | 1.48 | 2.37 | 0.0015 | 2.87 | 1.91 | 0.0016 |
| *optix>tkvRI* | -3.57 | 0 | 17.54 | 1.34 | 2.14 | 0.0028 | 5.35 | 3.56 | 0 |
| *optix>tkvRI+DADRI* | 1.99 | 0 | 20.92 | 1.16 | 1.86 | 0.0028 | 2.36 | 1.57 | 0.0212 |
| *optix>DADRI* | 1.39 | 0 | 22.50 | 0.93 | 1.50 | 0.0386 | 2.84 | 1.89 | 0.0096 |
| *optix>tkvRI+RHGRI* | 1.23 | 0.0084 | 23.01 | 1.18 | 1.90 | 0.0070 | 2.90 | 1.93 | 0.0417 |
| *BAR* | -4.60 | 0 | 16.52 | 1.63 | 2.62 | 0.0001 | 5.64 | 3.75 | 0 |
| *BAR:optix>RHGRI* | 0.85 | 0.2091 | 21.61 | 1.16 | 1.87 | 0.0072 | 2.68 | 1.78 | 0.0417 |
| *BAR:optix>DADRI* | 3.26 | 0.0017 | 21.17 | 1.09 | 1.74 | 0.0070 | 2.62 | 1.74 | 0.0293 |
