# Supplementary material for: Feedback control of organ size precision is mediated by BMP2-regulated apoptosis in the Drosophila eye
Source: PLoS Biol. 2024 Jan 30;22(1):e3002450. doi: 10.1371/journal.pbio.3002450 (PMC10826937; doi:10.1371/journal.pbio.3002450)
Supplement: S2 Table — Each genotype effect on %Apop median, their p.values, and the predicted median for each of them are shown (see main text, Materials and methods section). (PDF) [file pbio.3002450.s011.pdf]

Suppl. Table 1 to Figure 2. Analysis of genotype effect on % of apoptotic area in the anterior domain of the eye primordium (%Apop). Each genotype effect on %Apop median, their p.values and the predicted median for each of them are shown (See main text, Materials and Methods section).

	optix>+	optix>RHGRI	optix>tkvRI	optix>tkvRI+ DADRI	optix>DADRI	optix>tkvRI+ RHGRI
Effects	7.21	-5.15	31.21	-25.11	-5.25	-32.42
p.value	0	0	0.0028	0.0157	0	0.0020
Predicted by group (%)	7.21	2.06	38.41	8.05	1.95	0.84
