# Supplementary material for: Feedback control of organ size precision is mediated by BMP2-regulated apoptosis in the Drosophila eye
Source: PLoS Biol. 2024 Jan 30;22(1):e3002450. doi: 10.1371/journal.pbio.3002450 (PMC10826937; doi:10.1371/journal.pbio.3002450)

Suppl. Figure to Materials and Methods. Eye and head areas as measured in this work. (a,a') frontal (a) and occipital (a') focal planes, with the areas corresponding to the left eye outlined using the "lasso" tool of ImageJ. The area of the eye is the sum of both areas. (b) Area of the head, outlined using the lasso tool. The area of each eye was obtained as the sum of the area of the eye in the frontal and occipital planes (a + a').

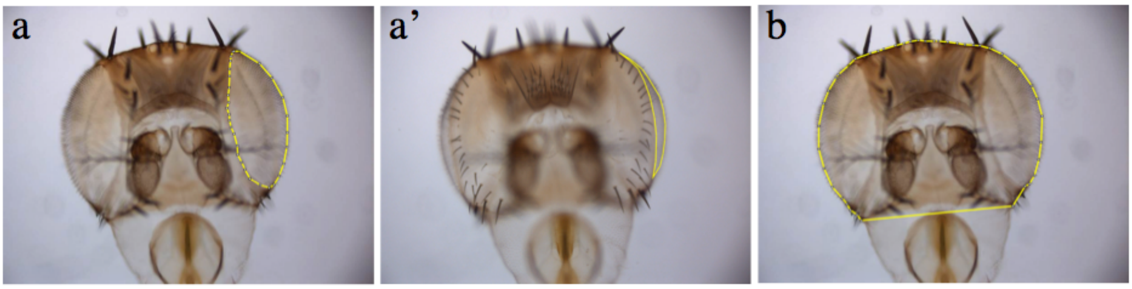

Supplement: S1 Materials — Eye and head areas as measured in this work. (a, a’) Frontal (a) and occipital (a’) focal planes, with the areas corresponding to the left eye outlined using the “polygon” tool of ImageJ. The area of the eye is the sum of both areas. (b) Area of the head, outlined using the “lasso” tool. The area of each eye was obtained as the sum of the area of the eye in the frontal and occipital planes (a + a’). (PDF) [file pbio.3002450.s012.pdf]
