# Supplementary material for: Feedback control of organ size precision is mediated by BMP2-regulated apoptosis in the Drosophila eye
Source: PLoS Biol. 2024 Jan 30;22(1):e3002450. doi: 10.1371/journal.pbio.3002450 (PMC10826937; doi:10.1371/journal.pbio.3002450)
Supplement: S1 Statistical Methods — (PDF) [file pbio.3002450.s013.pdf]

SUPPLEMENTARY STATISTICAL METHODS

% of proliferation area in the anterior region of the eye primordium: corrected estimation

If a disc has a large progenitor area undergoing apoptosis, using this area to calculate the mitotic rate (%prolif) will produce an underestimation of this rate –as apoptotic cells cannot undergo mitosis. While this effect is rightly neglected in the literature for control/wild type discs (as the normal apoptotic rate is low), in our experiments, where apoptosis can be very abundant, this fact needs to be taken into account. To do so, proliferation density has to be estimated according to the proliferation-competent (non-apoptotic) anterior region area (AV). Ideally, AV could be directly calculated as the difference between total and apoptotic areas for each disc and then proliferation density estimated as a ratio. Nevertheless, measurements of proliferation and apoptosis were taken on different eye imaginal disc. The apoptotic rate (expressed as the percentage of the progenitor area expressing the Dcp-1 apoptotic marker) depends on the genotype, so we first estimated the %AV for each genotype from Dcp-1 stained discs and then applied the correction to the non-corrected raw % of proliferation (measured as the percentage of the progenitor area expressing the mitotic marker PH3), as:

$$\%\text{prolif} = \%\text{prolif(raw)}/\%\text{AV}$$

Statistical analysis of GAL4 “dilution effect” and % of proliferation

To test the null hypothesis that the GAL4/UAS is equally efficient in driving UAS-targets regardless of whether there is one or two UAS sequences in the genotype, we compared the phenotypes of *optix>tkvRI* (one UAS sequence) against *optix>tkvRI + GFP* (two UAS sequences). Since *optix>tkvRI* flies have smaller eyes (rE) and larger FAi than controls, if there were a “GAL4 dilution” effect (the GAL4-driven expression of the *UAS-tkv-RNAi* would be diminished in the presence of a second *UAS*), a weaker phenotype (i.e. larger rE and smaller FAi) would be expected. If (δ) expresses this dilution, we would then expect for rE and FAi that:

$$\begin{cases} H_0\colon \delta(\text{rE}) = 0 \\ H_1\colon \delta(\text{rE}) > 0 \end{cases}$$

$$\begin{cases} H_0\colon \delta(\text{FAi}) = 0 \\ H_1\colon \delta(\text{FAi}) < 0 \end{cases}$$

For the analysis of the % proliferation we do not have information on the alternative hypothesis, and the real effect could be positive or negative. Therefore:

$$\begin{cases} H_0\colon \delta(\%\text{prolif}) = 0 \\ H_1\colon \delta(\%\text{prolif}) \neq 0 \end{cases}$$

FAi was analyzed instead of its signed version (sFAi) in order to estimate the BF (Bayes Factor) and to determine the degree of acceptance of H_0 (absence of GAL4 "dilution effect" on eye size precision).

Bayes factor associated with one tailed t-student test

The p-value is used as a decision threshold to solve hypothesis testing from a frequentist approach. However, this decision criterion is aimed at demonstrating that H_0 is false but it does not, on the contrary, allow us to affirm that H_0 is true, since the p-value cannot be interpreted as a measure of the strength with which we accept H_0: not being able to demonstrate the falsity of H_0 does not prove its veracity. Since our aim here is precisely this, to prove H_0, we propose an alternative, Bayesian approach to solve the hypothesis testing. The Bayes Factor constitutes a measure of strength or confidence with which to accept H_0 (Dienes, 2014). Bayes factor (BF) is defined as the odds ratio between of finding the observed data under H_1 and under H_0:

$$BF = \frac{P(H_1)}{P(H_0)}$$

With values of BF greater than 1 we will tend to accept that H_0 is false, and true otherwise.

Whereas parameter δ distribution under the null hypothesis is well defined, under the alternative one is not, leading to a battery of possible real values for the parameter δ and subsequent density functions (Bayarri et al., 2016). ttestBF function from BayesFactor library allows to calculate BF associated to one tailed t-student test (nullInterval = c(0, Inf)).

Detectable difference bars at 80% power

The power of a test is fundamental in hypothesis testing, especially when aiming to prove H_0 (Cohen, 1988). It is defined as the probability of correctly rejecting H_0 and, in a complementary way, the type II error is defined as the probability of erroneously not rejecting H_0. We cannot confirm the veracity of H_0, but for a pre-specified effect size, δ_0, and sample size the power of the test can be determined. If we do not know what δ_0 can be expected between the 2 groups, we can proceed in the opposite way: pre-setting the type II error, at 20% (Cohen, 1988), and for the sample size collected calculate what would be the minimum effect size that the test could detect, δ_k, so that we can judge a priori whether that capacity for discrimination is high enough to accept H_0 or whether the test could be missing substantial differences between groups, that is, an underpowered test.

Although the power of the test described above is classically a frequentist concept, we can transfer it to the field of Bayesian statistics if we establish a decision threshold based on the BF (Lakens, 2014) (1, as mentioned before), as is done with the p-value (typically 0.05). We followed a Montecarlo approach to estimate type II error in BF-based decision threshold for each comparison:

First, the sample empirical distributions are centered at the same mean and then shifted iteratively from 0 to the target value, δ_k, the one for which the type II error of our test reaches the 20% target (80% power). Then, for each given $\delta_i>0$, a resampling of size n (number of individuals in the original samples by group and sex) from the sample empirical distributions is repeated 1000 times (*remp* function from fishmethods package) and BF calculated in each iteration (ttestBF function mentioned above). This

simulation allows to calculate how many times BF was lower than 1, that is, what percentage of the times H_0 would be wrongly not rejected (type II error).
The target value reached, δ_k, is the minimum detectable differences between groups by the BF-based decision threshold. By multiplying the resultant δ_k by the pooled standard deviation of the evaluated variable in the groups compared, we can express the minimum detectable differences in that variable's original scale and plot them as bars integrated in the sample distribution violin plots. The bar starting point is set at the *optix>tkvRI* median and oriented towards the expected value of δ_k under the alternative hypothesis (i.e.positive for rE and negative for FAi). The same method was followed to calculate detectable differences bars in the % of proliferation plot but, since we could not predict the direction of the effect in this case, the minimal detectable differences were represented both above and below the mean.
These power bars can be understood as "If real differences between populations were larger than shown by bars the test would detect them 80% of the times".

Significance testing of coefficients in ΔFA model
Inter-individual Fluctuation asymmetry (FAn) and Fluctuation asymmetry differences (ΔFA) were defined as follows:

$$\mathrm{FAn} = \left|\frac{L_i}{H_i} - \frac{R_j}{H_j}\right| \; ; \; \Delta\mathrm{FA} = \mathrm{FAn} - \mathrm{FAi}$$

Where:

- i refers to the i-th individual
- j refers to random individual different from i

Due to FAn metrics (and ΔFA subsequently) for each group there are $n!^k$ possible combinations of randomized eye pairs, where n is the group size and K is the number of sex-segregated groups. This combinatorial leads to a more than $20!^{18}$ different fitted models for each of the metrics. In order to fix the problem, we used a bootstrap strategy: first, each left eye was randomly paired once and without replacement with any intragroup right eye. Then, a quantile regression model was fitted, and model coefficients were estimated (genotypic effects, See Main Statistical Methods). This resampling was repeated 1000 times. Thus, a distribution of coefficients was obtained, and 95% confidence intervals were estimated. Finally, p.values for significance testing of model coefficients were calculated also from its empirical distribution: probability of each coefficient of being smaller than 0 when coefficient estimation is positive and larger than 0 in the opposite situation.

Real and computational results comparison
One of the goals of this work was the implementation of a computational model of *Drosophila* eye development with which to calculate predicted eye size and precision as a function of variables regulating cellular proliferation, apoptosis and differentiation. These cellular processes can be experimentally manipulated by using targeted gene expression and attenuation methods (see Methods, main section). The computational model must yield results of eye size and precision obtained for different genotypes (dubbed here "real") after parameter fitting. The following figure shows the workflow for the validation of the computational model.

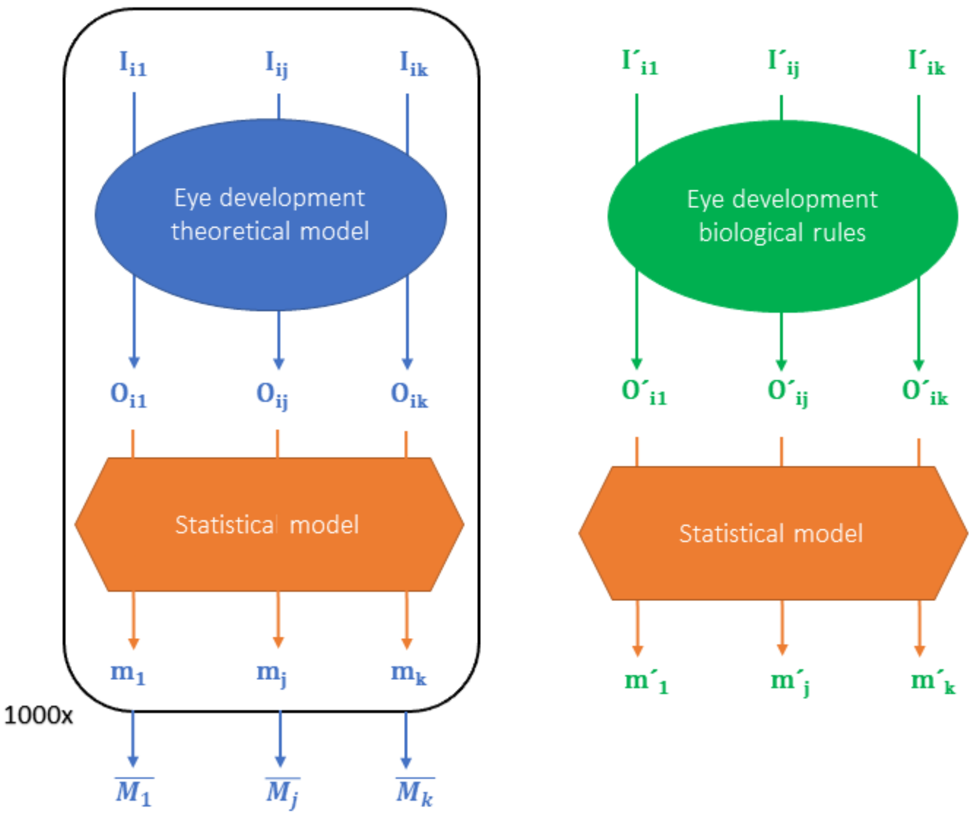

The *i*-th pair of eyes of each sample of size n is generated from the *j*-th specific combination of parameters or biological conditions (I*ij*; I´*ij*). These k conditions represent the inputs for the computational model and the biological system (the eye), respectively, and the outputs (Oij; O´ij) are values of rE and FAn. Then, the same statistical model (median regression) applied to computational and "real" (i.e. biological) data allow predicting the median of each output for each of k groups (mj; m´j). Medians predicted by quantile regression of the output values of the computational model and biological system (mj; m´j) could be directly compared. Having into account that there is a lower limit to repeat the sampling process in the computational model we followed a Montecarlo simulation strategy by reiterating the sampling 1000 times to reach the approximated distribution of Mj.

At this point, we can pose the hypothesis to be tested in order to demonstrate that the theoretical model predicts the same eye size and precision that the respective biological genotypes:

$$\begin{cases} H_0: M'_j \sim M_j \ \forall\, j \in [1, \mathrm{k}] \\ H_a: \exists\, j \mid M'_j \sim \mathrm{X} \neq M_j \end{cases}$$

Under the null hypothesis at 95% confidence, the following statements must be true:

$$\mathrm{m'j} \ \in \ (\mathrm{P}_{2,5}(\mathrm{Mj}), P_{97,5}(\mathrm{Mj})) \ \forall\, \mathrm{j} \ \in \ [1, \mathrm{k}]$$

It means that the biological rE and FAn medians belong in their respective theoretical median distributions and, conversely, that the theoretical model is able to explain the observed experimental results.
Because we found a small correlation between the eyes of the same individual (Suppl. Fig. 5 to Figure 1) that cannot be accounted for by the computational model, we decided to compare the empirical FAn median, rather than the median of FAi (which is computed using left and right eye metrics of each individual) with the computational FA median distribution.

References

Bayarri, M.J., Benjamin, D.J., Berger, J.O., Sellke, T.M., 2016. Rejection odds and rejection ratios: A proposal for statistical practice in testing hypotheses. J Math Psychol 72, 90-103.
Cohen, J., 1988. Statistical power analysis for the behavioural sciences. Hillsdale, N.J. : L. Erlbaum Associates.
Dienes, Z., 2014. Using Bayes to get the most out of non-significant results. Front Psychol 5, 781.
Lakens, D., 2014. The 20% Statistician: Power analysis for default Bayesian t-tests, http://daniellakens.blogspot.com/2016/01/power-analysis-for-default-bayesian-t.html.
