# Supplementary material for: Feedback control of organ size precision is mediated by BMP2-regulated apoptosis in the Drosophila eye
Source: PLoS Biol. 2024 Jan 30;22(1):e3002450. doi: 10.1371/journal.pbio.3002450 (PMC10826937; doi:10.1371/journal.pbio.3002450)
Supplement: S1 Mathematical Model — (PDF) [file pbio.3002450.s014.pdf]

MATHEMATICAL MODEL

DETERMINISTIC MODEL

We propose a model with three variables: G will be a measure of the width of the progenitor population; R_n a measure of the newly differentiated retinal cells, capable of producing Dpp; and R a measure of the cells undergoing terminal differentiation and patterning, Fig. 1. The process will finish when all cells have differentiated and R becomes a stationary value. We assume that Dpp production is proportional to R_n, the width of the region producing it. The effect of *hedgehog* (Hh) will be proportional to R. Progenitor cells can die through apoptosis, this process being repressed by Dpp. Experimental observations [1] indicate that the cell cycle becomes slower as the differentiated region of the eye becomes bigger: the doubling time of the progenitors is proportional to R. Denoting with c_0 the constant of proportionality, the rate of cell division per cell is:

$$\text{cell division rate} = \frac{\log(2)}{c_0 R}. \tag{1}$$

We will write our equations including this explicit factor $\log(2)/c_0 R$ multiplying every term; therefore this varying timescale will act as the time unit of our system, and apoptotic and differentiation rates will be expressed referred to it. In this way, our model will account for the decisions (proliferation, apoptosis, differentiation) taken by cells during the time interval corresponding to one cell cycle of the progenitors. Moreover, including the varying cell cycle duration Eq. (1) is a condition without which there is a strict experimental constraint that otherwise would be very difficult to reproduce by the model: the speed of the differentiation front is, for most of development, constant [1, 2].

Conceptually, this model can be written as a set of ordinary differential equations for the variables G, R_n and R, where the evolution of each variable depends on a number of processes:

$$\frac{dG}{dt} = \frac{\log(2)}{c_0 R}(\text{proliferation} - \text{apoptosis} - \text{initial\_differentiation}) \tag{2}$$

$$\frac{dR_n}{dt} = \frac{\log(2)}{c_0 R}(\text{initial\_differentiation} - \text{final\_differentiation}) \tag{3}$$

$$\frac{dR}{dt} = \frac{\log(2)}{c_0 R}(\text{final\_differentiation}). \tag{4}$$

$G(t)$, $R_n(t)$ and $R(t)$ are functions of time t, but for the sake of clarity we have dropped t from the notation.

Following previous models for cell proliferation [3–5], we denote the probability of a progenitor cell undergoing apoptosis along the duration of a cell cycle as the function $a(R_n)$: it is a decreasing function of R_n because Dpp inhibits apoptosis. Therefore, the apoptosis term in Eq. (2) is:

$$\text{apoptosis} = a(R_n)G. \tag{5}$$

The more progenitor cells, the more new cells we have in each cycle of cell division; however, only cells not undergoing apoptosis during a cell cycle can divide. Taking this into account, the proliferation term in Eq. (2) is:

$$\text{proliferation} = (1 - a(R_n))G. \tag{6}$$

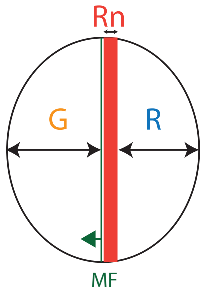

Figure 1: Schematic representation of the developing eye. To the left, progenitor cells undergo cell division, leading to the growth of the eye. In the central region, a narrow band of newly differentiated retinal cells produces Dpp. To the right, completely differentiated retinal cells will form the eye; also, they produce *hedgehog*. We denote the widths of each of these regions as: G (progenitor cells), R_n (newly differentiated retinal cells) and R (completely differentiated retinal cells). The morphogenetic furrow (MF) roughly coincides with the frontier between progenitors and newly differentiated retinal cells, and its progression occurs at approximately constant velocity throughout most of the process.

For the rate of initial differentiation, we have to distinguish two contributions. First, it has to saturate with G, because beyond certain point having larger G should not produce faster differentiation, since the region of G differentiation is limited to the range of action of R_n-produced Dpp, and only cells close to the G-R_n boundary differentiate. We model this spatial effect through a sigmoidal function $f(G)$; by imposing $f(0) = 0$ this function also ensures that when the progenitor population disappears, no new retinal cells can appear. In some sense, this limitation of the width of the progenitor cell region over which differentiation has an effect takes into account Dpp's range of action in this tissue. The second contribution to the rate of initial differentiation would be given by the activation of differentiation by *hedgehog*. We have also studied including a dependence of this rate on Dpp, but since it does not improve the results, for simplicity we do not use it. This does not exclude, however, that Dpp can play a role in this process. Hence, this contribution is an increasing function of R; we denote it as $h(R)$, resulting in:

$$\text{initial\_differentiation} = f(G)h(R). \tag{7}$$

Experimental observations of the width of the region of newly differentiated cells indicate that it stays almost constant throughout most of the process. We have monitored, during development, Hh signaling range, using as a read-out the active form of Ci (CiA), the nuclear transducer of the pathway. This range should be equivalent to the R_n domain, as R_n cells are induced by Hh signaling. The data does not show a significative trend over time in the width of this domain. This implies that cells exit the R_n state at roughly the same rate that they enter it. Therefore, the rate of cells differentiating from $G \to R_n$ has to be related to the rate of cells exiting the R_n state, $R_n \to R$. This can be visualized as cells in R_n having some sort of *timer* that triggers final differentiation into R at a certain time after becoming R_n. This time would correspond to the time needed for a recently recruited cell to undergo the gene expression changes required for its differentiation into

a Hh-producing cell. It is not our place here to discuss the possible mechanism behind this timer, although a plausible explanation could be a roughly constant range of Hh signaling coupled to the approximately constant velocity of the differentiation wavefront. The simplest way to model this is by making the rate of final differentiation also proportional to $h(R)$:

$$\text{final\_differentiation} = b\, h(R) R_n, \tag{8}$$

where b is a constant. This functional form implies that looking for a quasi-steady value of R_n, which is the condition $dR_n/dt \approx 0$, we would have:

$$R_n \approx f(G)/b, \tag{9}$$

which will be approximately constant as required if $f(G)$ is a saturated function of G, as expected during most of the process. Note that making the final differentiation rate proportional to $h(R)$ does not necessarily mean that R regulates this rate; rather, it is a simplified way to model the intrinsic *timer* in R_n cells that starts ticking when they undergo the initial $G \to R_n$ differentiation. When this timer runs off, R_n cells differentiate into R, and hence the rate of this final differentiation is proportional to the initial $G \to R_n$ differentiation. Also, it should be noted that in our simplified model, having R_n at a quasi-steady state and using it as a proxy for Dpp concentration means it is difficult to see the effects of Dpp purely in the dynamics of the model: that explains why we can neglect it in the initial_differentiation term, although biologically it might be a relevant factor. However, changing Dpp levels can still have a sizable effect on apoptotic probabilities.

To close the model definition, for the function modeling apoptosis we choose the Hill function:

$$a(R_n) \equiv \frac{a_0}{1 + (R_n/K)^r}, \tag{10}$$

where a_0 is the maximum apoptosis probability in the absence of Dpp, K is the value of R_n that halves the apoptosis probability, and r the exponent describing the cooperativity of this repression. We choose $r = 2$ to have enough nonlinearity in the Dpp feedback. The term $a(R_n)$ is a phenomenological model of how Dpp inhibits differentiation. In a fully spatial model, the concentration of Dpp would be position-dependent, but we would still need to introduce a function of this sort. In our current model, with no space, it should be understood in a mean-field sense.

For initial differentiation, we choose to model the saturation of differentiation rate with progenitor population using the Hill function:

$$f(G) \equiv \frac{G^w}{K_G^w + G^w}, \tag{11}$$

where K_G is the value of G that allows the differentiation $G \to R_N$ to proceed at half its maximum potential speed, and w the exponent defining the nonlinearity of the function $f(G)$. We choose $w = 1$ to ensure that for small G the rate of differentiation is linear in G, so:

$$f(G) \equiv \frac{G}{K_G + G}. \tag{12}$$

Note that K_G can be interpreted as a lengthscale of the width of the progenitor region that contributes to differentiation. Only when G is of the order or smaller than K_G, the differentiation rate

significantly decreases as the value of G diminishes. This function would not be necessary in a model with explicit modeling of space and morphogen gradients. Given that we don not have data to constrain such a model, $f(G)$ is a phenomenological way of inlcuding such effects.

To model the regulation of initial differentiation by *hedgehog* we choose the function:

$$h(R) \equiv b_r + q \frac{R^u}{{K_R}^u + R^u}, \quad (13)$$

where b_r is a basal differentiation rate independent of *hedgehog*, q the maximum differentiation rate activated by Hh, K_R the value of R for which this activation is half-saturated, and u the exponent defining the nonlinearity of this saturation. We choose $u = 2$ to have enough nonlinearity in the Hh feedback. The constant $b_r \neq 0$ is necessary to achieve a good fit between model and experimental data; it does not necessarily imply that differentiation can go on under the total absence of *hedgehog* or Dpp. Rather, it can model a situation where a small signal is enough to activate differentiation at a small rate, and only for higher morphogen values this rate will accelerate. The term $h(R)$ represents the effect of Hh promoting differentiation. In a spatial model Hh would be position dependent, but still a function of Hh of this sort would model the propensity of each cell to differentiate. As with $a(R_n)$, the function $h(R)$ has to be interpreted as a mean-field version of a spatially extended $h(Hh(x, y))$. Therefore, the constant K_r is not modeling a lengthscale, but a threshold of Hh activity. To maintain dimensional consistency with R, which itself has units of space, K_r is also expressed in terms of spatial dimensions. However, please note that K_r actually signifies the concentration of Hh at which its effect on differentiation reaches half saturation. Remember that we are taking R as a proxy for Hh levels. If we were to replace R by a proportional Hh concentration, the same proportionality constant would apply to K_r and convert it to its true dimension as a concentration. Therefore, contrary to K_G, the value of the phenomenological constant K_R should not be considered a meaningful lengthscale.

The regulatory logic implied by these definitions is shown in Fig. 2, and allows us to write the qualitative Eqs. (2)-(4) as the quantitative equations:

$$\frac{dG}{dt} = \frac{\log(2)}{c_0 R}((1 - a(R_n))G - a(R_n)G - f(G)h(R)) \quad (14)$$

$$\frac{dR_n}{dt} = \frac{\log(2)}{c_0 R}(f(G)h(R) - b\,h(R)R_n) \quad (15)$$

$$\frac{dR}{dt} = \frac{\log(2)}{c_0 R} b\,h(R)R_n. \quad (16)$$

Substituting the expressions for $a(R_n)$, $f(G)$ and $h(R)$, we have:

$$\frac{dG}{dt} = \frac{\log(2)}{c_0 R}\left(\left(1 - \frac{2a_0}{1 + (R_n/K)^2}\right)G - \left(b_r + q\frac{R^2}{{K_R}^2 + R^2}\right)\frac{G}{K_G + G}\right) \quad (17)$$

$$\frac{dR_n}{dt} = \frac{\log(2)}{c_0 R}\left(b_r + q\frac{R^2}{{K_R}^2 + R^2}\right)\left(\frac{G}{K_G + G} - b\,R_n\right) \quad (18)$$

$$\frac{dR}{dt} = \frac{\log(2)}{c_0 R}\left(b_r + q\frac{R^2}{{K_R}^2 + R^2}\right) b\,R_n. \quad (19)$$

As specified, these equations are deterministic. Next we will work out the way to include the effects of noise and fluctuations.

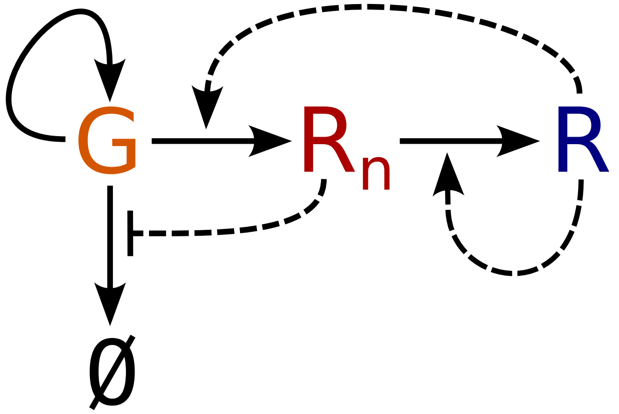

Figure 2: Regulatory logic of the model. Continuous arrows denote cellular transitions, dashed lines are positive (pointed arrow) or negative (blunt arrow) regulations of these transitions. Progenitor cells, G, can proliferate, differentiate into newly differentiated retinal cells, R_n, or die through apoptosis ($\emptyset$). Dpp produced by R_n inhibits apoptosis, *hedgehog* produced by finally differentiated retinal cells, R, promotes differentiation. R_n cells maturate into R cells.

PARAMETER VALUES

We have fitted parameter values to reproduce the dynamics of eye growth in *optix*$>$+, Figure 5 of the Main Text. Although Figure 5 does not show data for R_n, we have made independent observations of its typical value that have also been used to constrain the model. The parameter definitions and values are listed in the table below.

Table 1: Definition of the parameters in the model and values used for *optix*$>$+. Non-dimensional rate constants indicate proportionality factors to the cell division rate $\log(2)/c_0 R$.

c_0	progenitor's doubling time is $c_0 R$	0.1 h μm^{-1}
a_0	maximum apoptosis probability	0.3
K	value of R_n that halves the apoptosis probability	4.5 μm
b_r	basal differentiation rate independent of Hh	18
q	maximum differentiation rate activated by Hh	670
K_R	value of R for which Hh activation is half-saturated	190 μm
K_G	progenitor lengthscale contributing to differentiation	4.0 μm
b	final differentiation rate constant	0.04

To reproduce the experimental data in Figure 5 of the Main Tex, the initial condition we used for all simulations was $G = 32\mu$m, $R_n = 10\mu$m, $R = 5\mu$m. We have checked that our results are robust to adding reasonable amounts of noise to these initial conditions; moreover, the effects caused by this variability are difficult to distinguish from those of dynamic stochasticity (see below). Therefore, for simplicity, we do not discuss these observations.

The data we used to determine the parameter set, corresponding to a single condition, cannot univocally determine each individual parameter. This is a general characteristic of biological and physical models, termed *sloppiness* [6–8]. This is not a problem while not trying to reach conclu-

sions from individual parameter values: an equivalent fit with a different parameter set produces the same results in the range of validity of the model. We have indeed checked that our results are independent of the specific parameter set in the table above. In this sense, the parametrized deterministic model is a tool to understand the changes to it that lead to different conditions, and the mechanisms of modeling noise that reproduce the fluctuations observed experimentally.

MODELING RNAi TREATMENTS

We blocked apoptosis by driving, specifically anterior to the differentiation wavefront, a polycistronic RNAi against *reaper*, *hid* and *grim* (RHG), the three major pro-apoptotic genes in *Drosophila*: *optix>RHGRI* (see experimental Materials and Methods for details). The reduced apoptosis in *optix>RHGRI* is modeled using a lower value of a_0, the maximum apoptosis probability.

The Dpp pathway can be specifically attenuated in eye progenitors during development, by targeting the expression of an RNAi against the Dpp receptor *tkv* (thickveins) in these cells (*optix>tkvRI*). To confirm that results on *optix>tkvRI* result from the attenuation of the Dpp pathway, we drove expression of RNAis against *dad* (*daughters against-dpp*), a Smad molecule that acts as a feedback inhibitor of the pathway (*optix>DADRI*). Simultaneous use of both RNAis results in *optix>tkvRI+DADRI*.

To model these treatments we modify the parameter K, that sets the sensibility of apoptosis to the value of R_n: for *optix>tkvRI* we increase K, reducing the capability of R_n to inhibit apoptosis; for *optix>DADRI* we decrease K, making lower R_n values to have a stronger effect inhibiting apoptosis. Since we do not study serial treatments with different concentrations of tkvRI and DADRI, modeling with functions of their concentrations is unjustified and cannot be constrained. Moreover, since the effects of tkvRI and DADRI are surely nonlinear, a factor modeling the combination *optix>tkvRI+DADRI* is not necessarily a simple function of the factors $tkvRI$ and $DADRI$. Therefore, we also vary K by a factor that in principle can be greater or smaller than 1.

We also performed experiments combining RNAis targeting *RHG* and *tkv*: *optix>tkvRI+RHGRI*. Again, the combination of these two treatments may lead to non-trivial effects; in particular, the reduction of Dpp feedback by RNAi targeting *tkv* may lead to a reduction of the anti-apoptotic effect of the RNAi against RHG. Therefore, to model *optix>tkvRI+RHGRI* we change the parameter K by the same factor as in *optix>tkvRI*, but allow for a lower reduction of a_0 than in the *optix>RHGRI* case.

In the following table, we list the parameters used to model the effects on apoptosis of these different conditions:

Table 2: Parameters used to simulate different conditions.

condition	parameter	value
optix>RHGRI	a_0	0.011
optix>tkvRI	K	6.8μm
optix>DADRI	K	3.7μm
optix>tkvRI+DADRI	K	4.6μm
optix>tkvRI+RHGRI	K	6.8μm
optix>tkvRI+RHGRI	a_0	0.077

LINEAR STABILITY OF THE DETERMINISTIC MODEL

To understand the effects of the regulatory logic in Fig. 2, we perform a linear stability analysis of the deterministic model given by Eqs. (14)-(16). Only some generic properties of the functions $a(R_n)$, $f(G)$ and $h(R)$ will be necessary, so the conclusions of this analysis do not depend on the specific choices we have made for these functions. First we have to determine the fixed points of the dynamics of Eqs. (14)-(16), these are the points for which:

$$\frac{dG}{dt} = 0 \tag{20}$$

$$\frac{dR_n}{dt} = 0 \tag{21}$$

$$\frac{dR}{dt} = 0. \tag{22}$$

Assuming the Hh feedback $h(R)$ as a strictly positive function implies that necessarily any fixed point of the deterministic Eqs. (14)-(16) has to fulfill $R_n = 0$. In turn, given $a(R_n) < 1$ (as corresponds to a probability) and that $f(G)$ is a positive function with $f(0) = 0$ (there can be no initial differentiation when there are no progenitors), the other condition for a fixed point is $G = 0$. The variable R is not determined uniquely determined at the fixed point: it depends on the initial conditions. We denote the value of R when $G = R_n = 0$ as R^*, and it is always unstable, since any positive value of R_n will make it change. However, if we can ensure stability of the $G = 0$, $R_n = 0$ fixed point, this will not be a problem, since this fixed point is an *absorbing state*: once the G and R_n pools are depleted, there are no perturbations that can make new cells of these populations appear. So we only have to study the stability with respect to G and R_n, Eq. (14) and Eq. (15). For our choice of an initial differentiation rate that is a linear function of G for small G, the Jacobian of Eqs. (14–15) evaluated at the fixed point $G = 0$, $R_n = 0$ and $R = R^*$ is:

$$J = \begin{pmatrix} \frac{\partial \dot{G}}{\partial G} & \frac{\partial \dot{G}}{\partial R_n} \\ \frac{\partial \dot{R}_n}{\partial G} & \frac{\partial \dot{R}_n}{\partial R_n} \end{pmatrix} = \frac{\log(2)}{c_0 R^*} \begin{pmatrix} 1 - 2a(0) - f'(0)h(R^*) & 0 \\ f'(0)h(R^*) & -bh(R^*) \end{pmatrix}, \tag{23}$$

where $f'(0)$ is $\partial f / \partial G$ evaluated at $G = 0$. The eigenvalues of J are its diagonal entries,

$$\lambda_1 = 1 - 2a(0) - f'(0)h(R^*) \tag{24}$$

$$\lambda_2 = -bh(R^*). \tag{25}$$

The condition for stability of the fixed point is that these eigenvalues are negative. For a positive function $h(R)$, λ_2 automatically fulfills the condition; from λ_1 we obtain the stability condition:

$$2a(0) + f'(0)h(R^*) > 1. \tag{26}$$

It can be readily seen that increasing values of $a(0)$, $f'(0)$ and $h(R^*)$ favor stability: higher apoptosis probability for low Dpp, faster rate of initial differentiation and strong Hh feedback favoring initial differentiation contribute to the stability of the fixed point.

For our specific choices of $a(R_n)$, $f(G)$ and $h(R)$ in Eqs. (17–18), we have $a(0) = a_0$ and $f'(0) = 1/K_G$, yielding the Jacobian:

$$J = \frac{\log(2)}{c_0 R^*} \begin{pmatrix} 1 - 2a_0 - \left(b_r + q\frac{R^{*2}}{{K_R}^2 + R^{*2}}\right)/K_G & 0 \\ \left(b_r + q\frac{R^{*2}}{{K_R}^2 + R^{*2}}\right)/K_G & -b\left(b_r + q\frac{R^{*2}}{{K_R}^2 + R^{*2}}\right) \end{pmatrix}, \tag{27}$$

The stability condition now reads:

$$2a_0 + \left(b_r + q \frac{R^{*2}}{{K_R}^2 + R^{*2}} \right) / K_G > 1, \tag{28}$$

cleanly displaying that Dpp regulates stability through apoptosis, and Hh through differentiation. Experimental measurements of the apoptotic area in differentiated eyes show that it is around 40% in conditions in which apoptosis is not repressed. This would imply a value of $a_0 \approx 0.4$, meaning that the possibility of apoptosis, just by itself, is close to be enough to ensure stability of the fixed point, since $2a_0 \approx 0.8$ is close to 1.

STOCHASTIC MODEL

In order to introduce the effect of fluctuations, it will be convenient to rewrite the model in Eqs. (14)-(16) using a vector notation. Defining $X(t)$ as the vector

$$X(t) = \begin{pmatrix} G(t) \\ R_n(t) \\ R(t) \end{pmatrix}, \tag{29}$$

where t is time, we can write:

$$dX = F(X(t))dt, \tag{30}$$

where $F(X(t))$ is the so-called drift vector:

$$F(X) = \frac{\log(2)}{c_0 R} \begin{pmatrix} (1 - a(R_n))G - a(R_n)G - f(G)h(R) \\ f(G)h(R) - b\,h(R)R_n \\ b\,h(R)R_n \end{pmatrix}. \tag{31}$$

A standard way to include fluctuations in this kind of model, interpreting the different transition rates in a sense similar to propensities of chemical reactions, is to formulate the model as a system of Langevin equations [9,10]. Langevin equations are a type of stochastic differential equation (SDE) that, under certain conditions, are reasonable approximations of the stochastic dynamics of a set of chemical reactions or population. The main microscopic limits of this approximation translate, at an observational level, in the fact that the Langevin approximation breaks down when the noise distributions are multimodal [11]. In our work, the experimental results show noise distributions with a single mode, Figures 1 and 4 of the main text, therefore allowing the use of Langevin dynamics to model them. The noise term in an SDE can be interpreted mathematically in two different ways: the Itô or the Stratonovich sense. The distinction is very technical and out of the scope of our discussion: in our case, the noise has to be interpreted in the Itô sense [9,10]. The Langevin equation associated to the model in Eq. (30) is:

$$dX = F(X(t))dt + M(X(t))dW \tag{32}$$

where W is a vector of the four independent Wiener processes [9], representing the fluctuations associated to the four kind of processes in the model (proliferation, apoptosis, initial differentiation and final differentiation), and $M(X(t))$ is the diffusion matrix encoding the different contributions

to the noise. This noise that originates from the stochasticity of birth, death, and interaction processes in a population is usually termed *demographic noise*.

The diffusion matrix corresponding to the model with drift vector given by Eq. (31) is [10]:

$$M(X) = \sqrt{\frac{\log(2)}{c_0 R}} \begin{pmatrix} \sqrt{\frac{(1-a(R_n))G}{V_p}} & \sqrt{\frac{a(R_n)G}{V_a}} & -\sqrt{\frac{f(G)h(R)}{V_i}} & 0 \\ 0 & 0 & \sqrt{\frac{f(G)h(R)}{V_i}} & -\sqrt{\frac{b\,h(R)R_n}{V_f}} \\ 0 & 0 & 0 & \sqrt{\frac{b\,h(R)R_n}{V_f}} \end{pmatrix}, \tag{33}$$

where the V parameters determine the noise strength associated to each process: V_p is proportional to the inverse of the noise strength related to proliferation, V_a to apoptosis, V_i to the initial differentiation $G \to R_n$ and V_f to the final differentiation $R_n \to R$. Note that this matrix is non-diagonal: the fluctuations of different variables (rows of the matrix) that come from the same processes (columns of the matrix) are related. The random fluctuations are encoded in the stochastic vector dW that multiplies this matrix, $M(X)$ modulates those random numbers to produce the effect of the noise on each variable. Considering:

$$dW = \begin{pmatrix} dW_p \\ dW_a \\ dW_i \\ dW_f \end{pmatrix}, \tag{34}$$

as the *random rolls of dice* at each infinitesimal of time dt for the processes of proliferation, apoptosis, initial differentiation, and final differentiation, respectively, the vector $M(X)dW$ gives us the stochastic part of the equations of each of our three variables G, R_n and R:

$$M(X)dW = \sqrt{\frac{\log(2)}{c_0 R}} \begin{pmatrix} \sqrt{\frac{(1-a(R_n))G}{V_p}}dW_p + \sqrt{\frac{a(R_n)G}{V_a}}dW_a - \sqrt{\frac{f(G)h(R)}{V_i}}dW_i \\ \sqrt{\frac{f(G)h(R)}{V_i}}dW_i - \sqrt{\frac{b\,h(R)R_n}{V_f}}dW_f \\ \sqrt{\frac{b\,h(R)R_n}{V_f}}dW_f \end{pmatrix},$$

Note the opposite sign of the expressions multiplying dW_i: a fluctuation in initial differentiation affects in the same quantity but opposite sign the dynamics of G and R_n. The same applies for fluctuations associated to final differentiation, dW_f: they have the same value but opposite sign for R_n and R.

Stochastic equations have been numerically integrated using the Euler-Maruyama method from the function sde_euler in SDETools, a Matlab toolbox for the numerical solution of stochastic differential equations [12]. The option 'NonNegative' is set to 'yes', to ensure that the noise does not drive to negative values variables that are positive by definition.

Our model uses continuous variables, as opposed to the discrete nature of cells. To avoid that fluctuations unrealistically keep growth going when the values of G or R_n are well below the size of a cell, we set a threshold of 0.1μm, below which we make these variables zero. The multiplicative character of noise ensures that then fluctuations also disappear.

For the V parameters determining noise strength, we have used the values in the following table:

Table 3: Parameters used to define the noise strength associated to each process. For *optix>+* we have multiplied these values by a factor 6, making the noise weaker.

V_p	inverse of the noise strength related to proliferation	100
V_a	inverse of the noise strength related to apoptosis	1.0
V_i	inverse of the noise strength related to initial differentiation	6000
V_f	inverse of the noise strength related to final differentiation	6000

The exact value of the parameters is not that relevant: the key observation is that, to reproduce the high asymmetry observed between eyes in *optix>tkvRI*, apoptosis has to be a process much noisier than all others. From an intuitive point of view, this makes sense: differentiation is driven by well organized morphogens, but apoptosis in our model cells is just a probability for each cell to die randomly during a cell cycle.

Figure 6 of the Main Text shows that our model does an excellent job reproducing the eye sizes for different conditions. The fit of the FAi in different conditions could also have been almost perfectly good if we had allowed for different values of the noise parameters in each condition. We have chosen not to do so and not to fit the FAi with different V parameters for different conditions; instead, we use a single parameter set for the noise and let the changes in apoptotic mechanics in each condition to be the sole drivers of differences in asymmetry. However, conditions like *optix>tkvRI+DADRI* rescue the eye size but present higher asymmetry values than *optix>+*. The *optix>RHGRI* condition, that we have modeled as a reduction in the probability of apoptosis, is also more noisy than *optix>+*. This is difficult to reconcile with the observation, Figure 6 of the Main Text, that *optix>+* presents lower FAi than any of these conditions. Since second-order effects of these conditions on the noise are out of the scope of our model, for simplicity to approximate the FAi for *optix>+* we have multiplied all the V parameters in Table 3 by a factor 6, therefore reducing the noise for this condition.

NOISE STRENGTH OF THE DIFFERENT PROCESSES

Our determination of the parameter values of the noise strength associated with each process, Table 3, shows that of all the processes included in the model, apoptosis has to be the noisiest (V_a is small compared to other noise parameters) in order to explain the high asymmetry between eyes observed when apoptosis is not repressed. To illustrate how this is indeed the case, we show the comparison of simulation and experimental results for eye size and asymmetry, Figure 3. We have then studied the effect of having weaker noise associated with apoptosis, Figure 4: in this case FAi obtained from simulations for *optix>tkvRI* is similar to other conditions. Strong noises in the other processes while keeping apoptotic noise low, Figures 5–7, produce high FAi for all conditions, not discriminating *optix>tkvRI* from the other conditions. From the nature of the conditions we have studied, where apoptosis is primarily affected, we expected that the high asymmetry of the *optix>tkvRI* condition would be associated with apoptotic noise, while a strong contribution to noise of other processes would not discriminate this condition and produce high levels of asymmetry in all the conditions we have simulated. Our simulations support this picture.

For a more systematic understanding of noise effects, we have calculated the Fluctuating

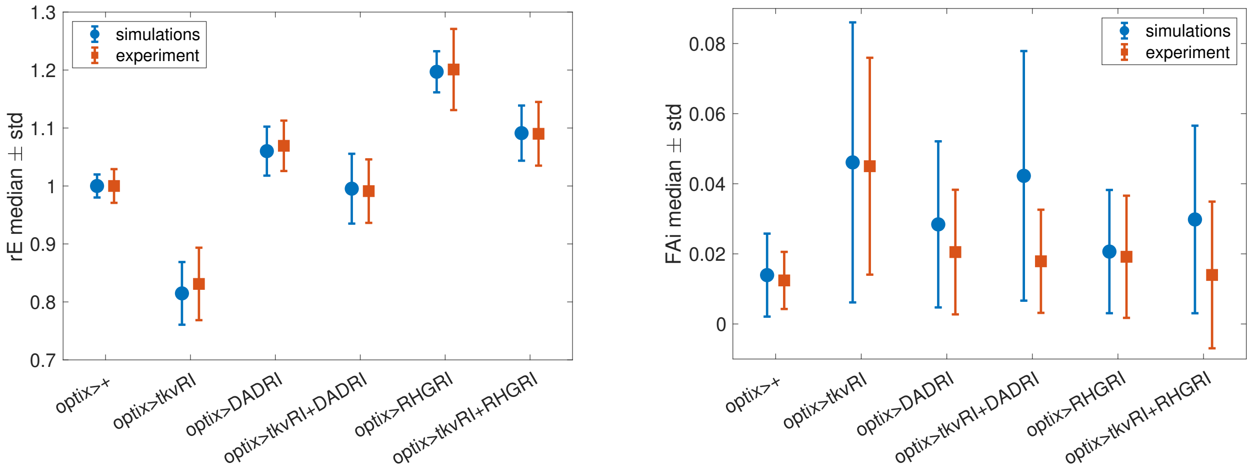

Figure 3: Standard noise (strong apoptotic noise): comparison for different conditions, as indicated, of simulation and experimental results. Simulations of 100 eyes with parameters in Tables 1–3. Left: eyes size, normalized to allow comparison of simulation and experiment. rE is the average intraindividual eye size. Right: eyes asymmetry. FAi is the intraindividual factor of asymmetry. Virtual *flies* with two eyes have been created by making all possible pairs of the 100 eyes.

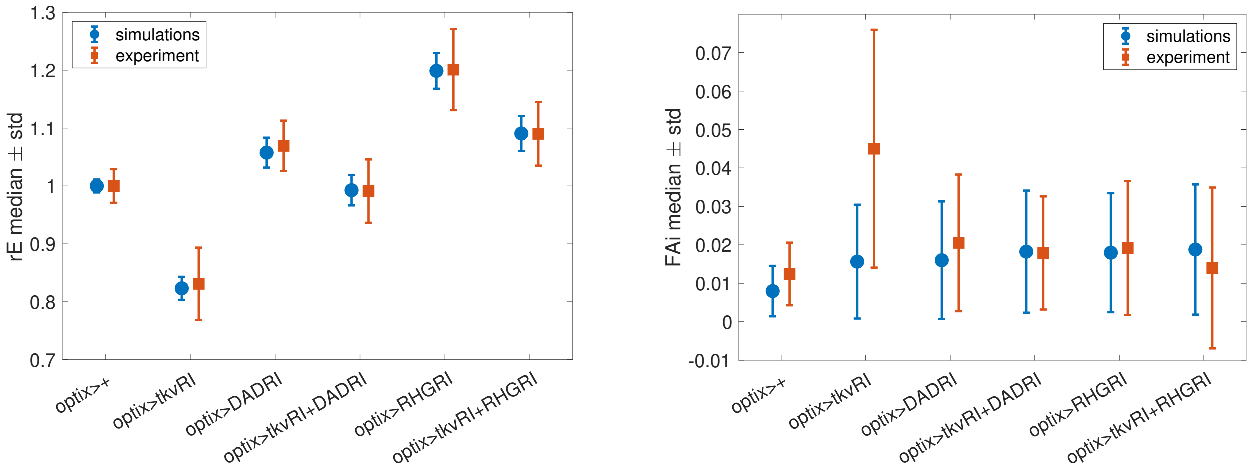

Figure 4: Weak apoptotic noise: comparison for different conditions, as indicated, of simulation and experimental results. Simulations of 100 eyes with $V_a = 100$, other parameters as in Tables 1–3. Left: eyes size, normalized to allow comparison of simulation and experiment. rE is the average intraindividual eye size. Right: eyes asymmetry. FAi is the intraindividual factor of asymmetry. Virtual *flies* with two eyes have been created by making all possible pairs of the 100 eyes.

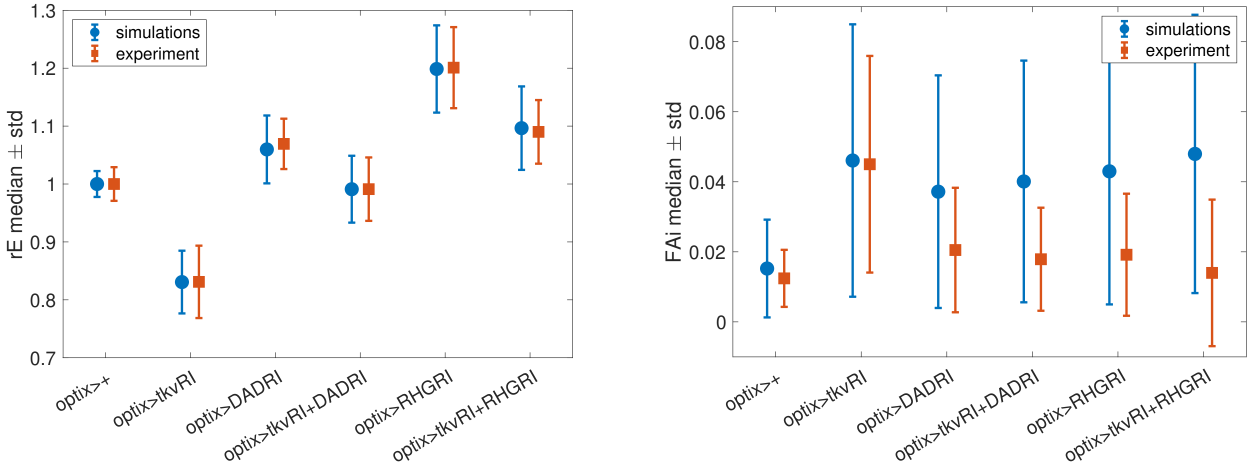

Figure 5: Weak apoptotic noise and strong proliferation noise: comparison for different conditions, as indicated, of simulation and experimental results. Simulations of 100 eyes with $V_a = 100$, $V_p = 20$, other parameters as in Tables 1–3. Left: eyes size, normalized to allow comparison of simulation and experiment. rE is the average intraindividual eye size. Right: eyes asymmetry. FAi is the intraindividual factor of asymmetry. Virtual *flies* with two eyes have been created by making all possible pairs of the 100 eyes.

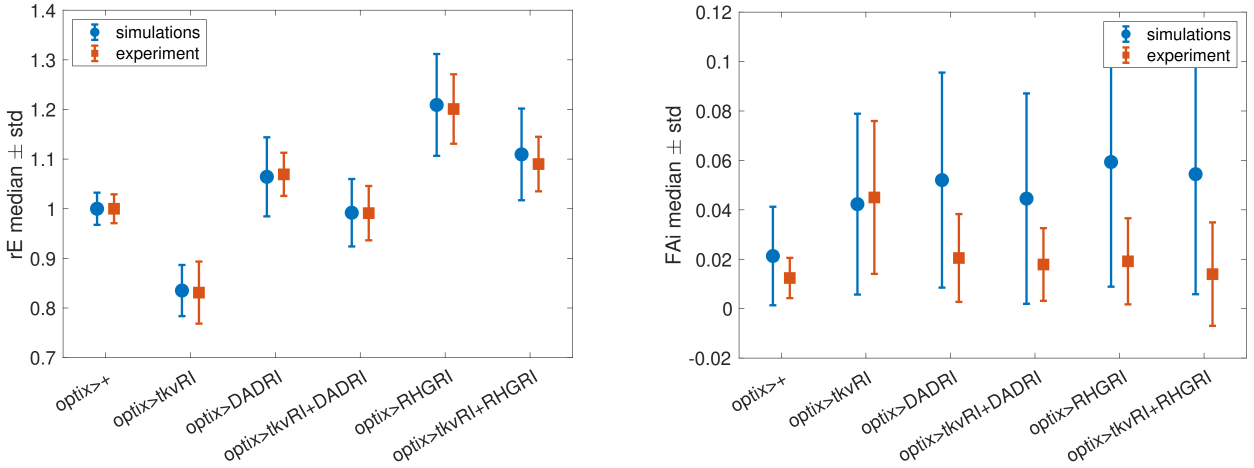

Figure 6: Weak apoptotic noise and strong initial differentiation noise: comparison for different conditions, as indicated, of simulation and experimental results. Simulations of 100 eyes with $V_a = 100$, $V_i = 15$, other parameters as in Tables 1–3. Left: eyes size, normalized to allow comparison of simulation and experiment. rE is the average intraindividual eye size. Right: eyes asymmetry. FAi is the intraindividual factor of asymmetry. Virtual *flies* with two eyes have been created by making all possible pairs of the 100 eyes.

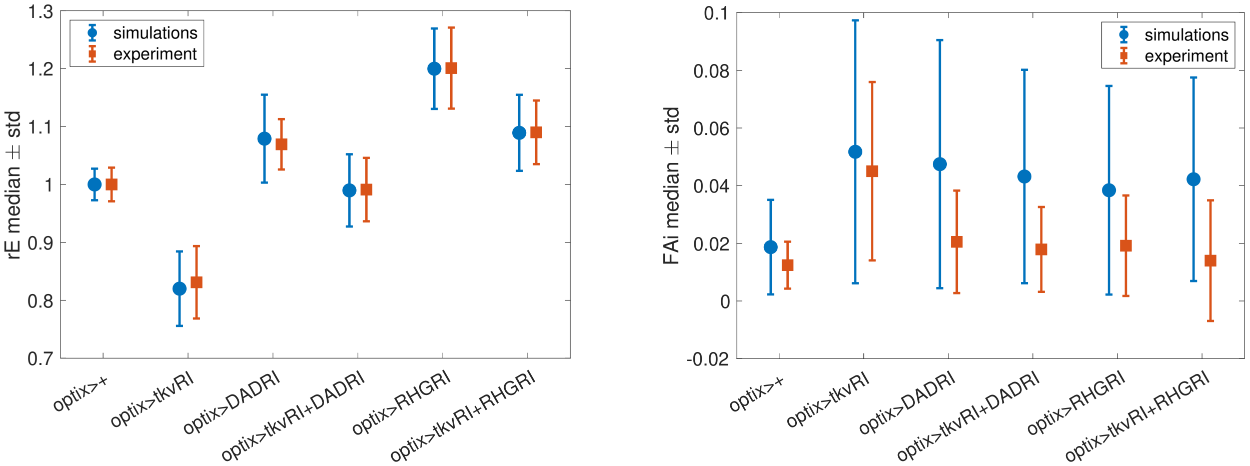

Figure 7: Weak apoptotic noise and strong final differentiation noise: comparison for different conditions, as indicated, of simulation and experimental results. Simulations of 100 eyes with $V_a = 100$, $V_f = 4$, other parameters as in Tables 1–3. Left: eyes size, normalized to allow comparison of simulation and experiment. rE is the average intraindividual eye size. Right: eyes asymmetry. FAi is the intraindividual factor of asymmetry. Virtual *flies* with two eyes have been created by making all possible pairs of the 100 eyes.

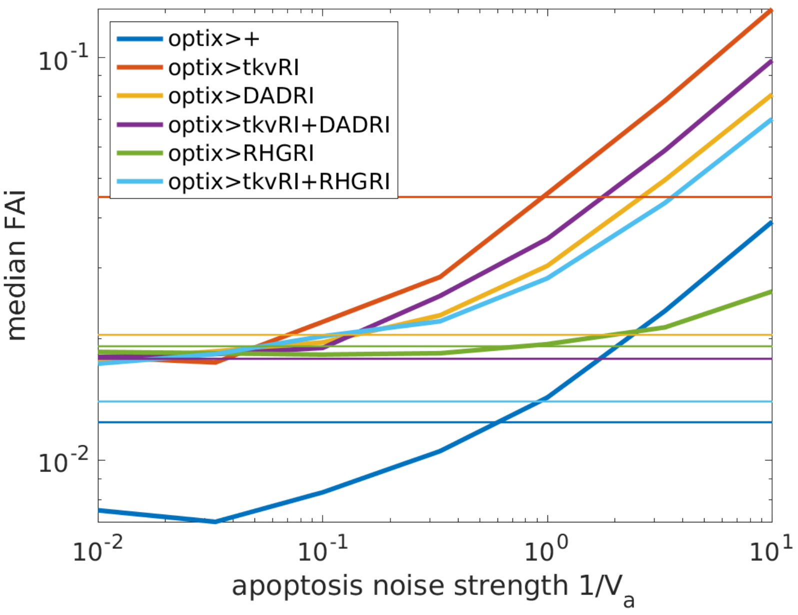

Figure 8: Fluctuating asymmetry index, FAi, of different conditions, as indicated, for a range of values of the apoptotic noise strength. Simulations of 1000 eyes, parameters as in Tables 1–3. Thin horizontal lines are FAi experimental values for the corresponding condition. Virtual *flies* with two eyes have been created by making all possible pairs of the 200 eyes.

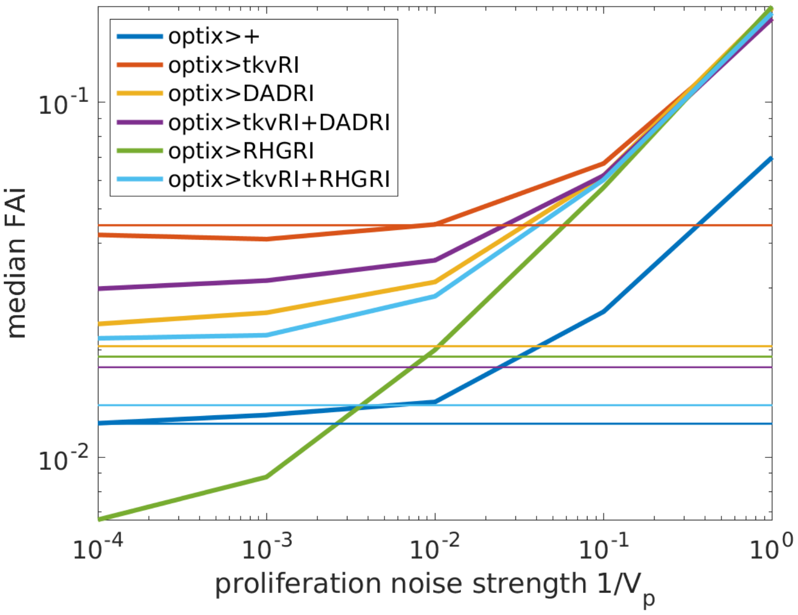

Figure 9: Fluctuating asymmetry index, FAi, of different conditions, as indicated, for a range of values of the proliferation noise strength. Simulations of 1000 eyes, parameters as in Tables 1–3. Thin horizontal lines are FAi experimental values for the corresponding condition. Virtual *flies* with two eyes have been created by making all possible pairs of the 200 eyes.

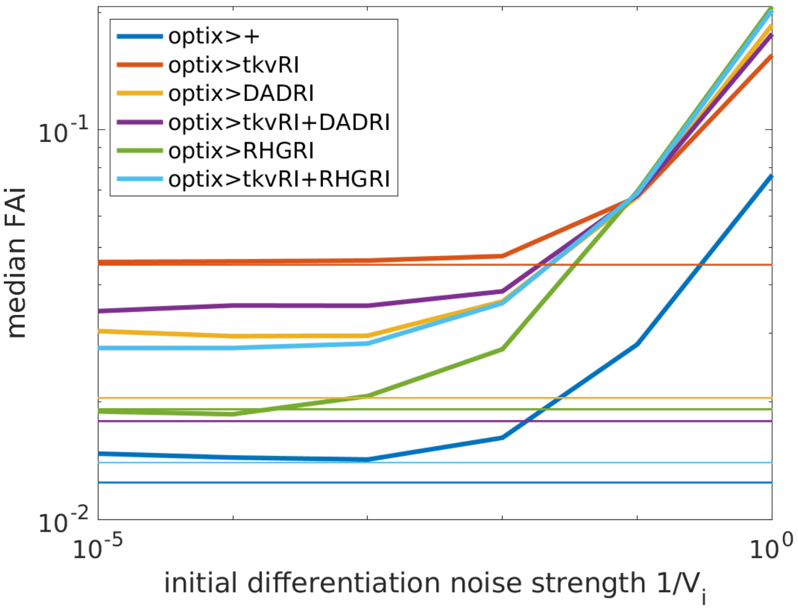

Figure 10: Fluctuating asymmetry index, FAi, of different conditions, as indicated, for a range of values of the initial differentiation noise strength. Simulations of 1000 eyes, parameters as in Tables 1–3. Thin horizontal lines are FAi experimental values for the corresponding condition. Virtual *flies* with two eyes have been created by making all possible pairs of the 200 eyes.

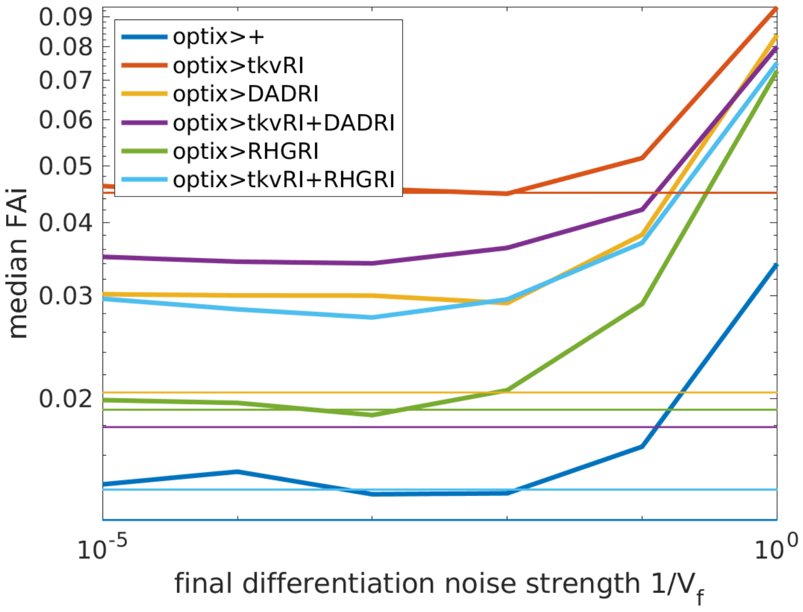

Figure 11: Fluctuating asymmetry index, FAi, of different conditions, as indicated, for a range of values of the final differentiation noise strength. Simulations of 1000 eyes, parameters as in Tables 1–3. Thin horizontal lines are FAi experimental values for the corresponding condition. Virtual *flies* with two eyes have been created by making all possible pairs of the 200 eyes.

Asymmetry index, FAi, for a range of values of each of the noise strength parameters while keeping the others constant, Figures 8–11. In Figure 8 we see that for apoptotic noise strength the asymmetry is roughly the same in all conditions (please recall that for *optix*$>$*+* we have introduced a noise reducing factor). For high enough apoptotic noise the condition with enhanced apoptosis, *optix*$>$*tkvRI*, systematically presents higher asymmetry than any other condition (and please observe that the vertical axis is logarithmic). Figures 9–11 show that, once the apoptotic noise rate V_a is fixed at a value producing strong noise, low noise strengths of the other mechanisms are highly irrelevant for setting the values of FAi. Once those noises become strong enough, they tend to make all conditions equally asymmetric (recall again the noise reducing factor for *optix*$>$*+*), therefore showcasing that strong apoptotic noise is necessary for the qualitative observation of high asymmetry in the high apoptosis *optix*$>$*tkvRI* condition.

SENSITIVITY ANALYSIS

To asses the robustness of our results to parameter values, we have performed a sensitivity analysis [13, 14]. In our study, we characterize the sensitivity S_{YX} of the measurable variable Y in relation to variations in the parameter X as:

$$S_{YX} = \frac{\partial \log Y}{\partial \log X} \tag{35}$$

This metric is assessed at a specific location within the parameter domain. The logarithmic differ-

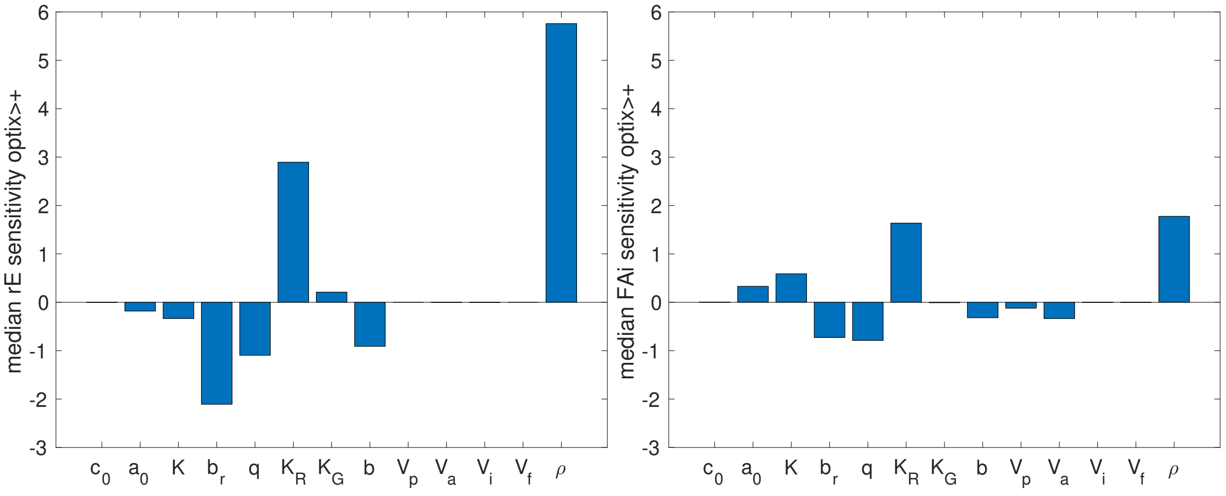

Figure 12: Sensitivity of the medians of rE (left panel) and FAi (right panel), calculated from 1000 realizations, with respect to 10% changes in the indicated parameter values. This analysis is computed at the point in parameter space used to describe the *optix>+* condition, Tables 1 and 3. Analysis of other conditions show similar results.

ential outlined above can be reformulated as:

$$S_{YX} = \frac{dY/Y}{dX/X} \tag{36}$$

For minor fluctuations in the parameter X, Eq. (36) can be approximated by the proportion of relative changes, expressed as:

$$S_{YX} = \frac{\Delta Y/Y}{\Delta X/X} \tag{37}$$

The model's sensitivity to shifts in the parameter X is ascertained by examining the chosen variable Y at two distinct points: the reference point X_0 and $X = X_0 + \Delta X$. Therefore, S_{YX} is defined as the quotient of the percentual variation in Y to the percentual variation in X. To determine senistivity values, we have used values of ΔX corresponding to 10% of the value of X_0. Since our model is stochastic, we have run 1000 simulations and determined the sensitivities of the medians of rE, the average intraindividual eye size, and FAi, the intraindividual factor of asymmetry. The results for medians of 100 simulations are virtually indistinguishable, showing that the caluclation has converged.

Figure 12 shows the results of our sensitivity analysis. Together to the parameters in Tables 1 and 3, we were interested in calculating the sensitivity to the proliferation rate. Since we had defined the timescales of all paramaters with respect to it, we have to introduce an explicit parameter that only affects proliferation rate. This is the parameter ρ in the following version of the drift equation of our model:

$$F(X) = \frac{\log(2)}{c_0 R} \begin{pmatrix} \rho(1 - a(R_n))G - a(R_n)G - f(G)h(R) \\ f(G)h(R) - b\,h(R)R_n \\ b\,h(R)R_n \end{pmatrix}. \tag{38}$$

This non-dimensional parameter has a value of $\rho = 1$, therefore the sensitivity analysis is donde comparing it to the results with a 10% change, corresponding to a value of $\rho' = 1.1$. Unsurprisingly, we find that this is the parameter to which the eye size is most sensitive. However, the intraindividual factor of asymmetry is much less sensitive to changes in growth rate: a trend for bigger eyes naturally allows for larger variations, but the resulting fluctuations in eye size are much constrained. This is a general property: Figure 12 shows that our model is robust to parameter values, and for those parameters with a higher sensitivity of the eye size, namely the proliferation rate ρ and the parameters K_R and b_r defining the initial differentiation rate, the corresponding sensitivity of the assymetry factor is sensibly lower. In particular, the model is very insensitive to small variations in the parameters determining noise strength, showing that our conclusions are in not dependent on a fine tuning of these parameters. The effects of large variations in the noise parameters was discussed in the previous section.

SUPPLEMENTARY REFERENCES

[1] Wartlick O, Jülicher F, Gonzalez-Gaitan M. Growth control by a moving morphogen gradient during Drosophila eye development. Development. 2014; 141:1884–1893. https://doi.org/10.1242/dev.105650

[2] Vollmer J, Fried P, Sánchez-Aragón M, Lopes CS, Casares F, Iber D. A quantitative analysis of growth control in the Drosophila eye disc. Development. 2016; 143:1482–1490. https://doi.org/10.1242/dev.129775

[3] Lander AD, Gokoffski KK, Wan FYM, Nie Q, Calof AL. Cell lineages and the logic of proliferative control. PLOS Biol. 2009; 7:e1000015. https://doi.org/10.1371/journal.pbio.1000015

[4] Lo WC, Chou CS, Gokoffski KK, Wan FYM, Lander AD, Calof AL, et al. Feedback regulation in multistage cell lineages. Math Biosci Eng. 2009; 6:59–82. https://doi.org/10.3934/mbe.2009.6.59

[5] Buzi G, Lander AD, Khammash M. Cell lineage branching as a strategy for proliferative control. BMC Biol. 2015; 13:13. https://doi.org/10.1186/s12915-015-0122-8

[6] Gutenkunst RN, Waterfall JJ, Casey FP, Brown KS, Myers CR, Sethna JP. Universally sloppy parameter sensitivities in systems biology models. PLOS Comput Biol. 2007; 3:e189. https://doi.org/10.1371/journal.pcbi.0030189

[7] Machta BB, Chachra R, Transtrum MK, Sethna JP. Parameter space compression underlies emergent theories and predictive models. Science. 2013; 342:604–607. https://doi.org/10.1126/science.1238723

[8] Transtrum MK, Machta BB, Brown KS, Daniels BC, Myers CR, Sethna JP. Perspective: Sloppiness and emergent theories in physics, biology, and beyond. J Chem Phys. 2015; 143:010901. https://doi.org/10.1063/1.4923066

[9] Gardiner CW. Handbook of stochastic methods for physics, chemistry and the natural sciences. vol. 13 of Springer Series in Synergetics. 3rd ed. Berlin: Springer-Verlag; 2004.

[10] Gillespie DT. The chemical Langevin equation. J Chem Phys. 2000; 113:297–306. https://doi.org/10.1063/1.481811

[11] Ham L, Coomer MA, Stumpf MP. The chemical Langevin equation for biochemical systems in dynamic environments. J Chem Phys. 2022; 157:094105. https://doi.org/10.1063/5.0095840

[12] Horchler A. SDETools (https://github.com/horchler/SDETools), GitHub. Retrieved March 15; 2023.

[13] Savageau MA. Parameter sensitivity as a criterion for evaluating and comparing the performance of biochemical systems. Nature. 1971; 229:542–544. https://doi.org/10.1038/229542a0

[14] Muñoz-García J, Ares S. Formation and maintenance of nitrogen-fixing cell patterns in filamentous cyanobacteria. Proc Natl Acad Sci U S A. 2016; 113:6218–6223. https://doi.org/10.1073/pnas.1524383113
