# Supplementary material for: Feedback control of organ size precision is mediated by BMP2-regulated apoptosis in the Drosophila eye
Source: PLoS Biol. 2024 Jan 30;22(1):e3002450. doi: 10.1371/journal.pbio.3002450 (PMC10826937; doi:10.1371/journal.pbio.3002450)
Supplement: Attachment — Submitted filename: Rebuttal.pdf [file pbio.3002450.s017.pdf]

Rebuttal

First of all, we would like to sincerely thank the reviewers for their thorough analysis of our paper and their comments and suggestions. In what follows, we address all the points they raised.

Rev. 1:

The manuscript by Casares, Ares and colleagues uses the Drosophila eye as model system and combines genetic manipulations and mathematical modelling to identify a role of Dpp in controlling eye size by blocking apoptosis in progenitor cells, to propose a role of apoptosis in reducing size variability and to generate a theoretical model to show how this newly identified mechanism contributes to reducing such variability. I am an experimentalist that appreciates mathematical modeling of biological processes but without sufficient background to review this part of the manuscript in a thorough manner. I have several issues with respect to the experimental part and their conclusions related to experiments and modeling that should be addressed to demonstrate and reinforce their proposal. I have only some minor comments to reinforce the role of Dpp in controlling eye size by reducing apoptosis (see at the end of the list below). However, I have major issues with the implication of Dpp and apoptosis in size precision. The two major comments listed below are fundamental to demonstrate their proposal.

Major (essential) comments:

(1) Authors claim that in all their experimental conditions (eg. different genetic manipulations to block apoptosis or reduce or activate Dpp signaling) size precision is lost as a result of increased variability and from these observations they propose that apoptosis plays a major role in the process. Is not the reduction in size precision a simple consequence of the developmental system being far away from the wild type (most probably, the most stable and precise) condition?

Have the authors analyzed other unrelated mutant conditions where there is a clear effect on eye size and/or patterning but without affecting size precision? I think this should be addressed by the authors to avoid any trivial explanation to their results and model.

R1: The reviewer is making a point that was raised by Waddington many years ago: "It is a very general observation (…) that the wild type of an organism, that is to say, the form which occurs in Nature under the influence of natural selection, is much less variable in appearance than the majority of mutant races" (C.H. Waddington, 1942). There are two assumptions under this statement. First, that genetic mechanisms must exist to "buffer" or "canalize" the wild type phenotype. The second, which is not explicit in the original paper, is that phenotype and its variation are mechanistically linked, such that the wild type genotype, which dictates the phenotype (in our case, eye size) also minimizes its variability. This latter is, in principle, at odds with the fact that phenotypes evolve (in the case, for example, of eye size in flies, strikingly so). This evolvability demands that the mean phenotype may vary while still remaining canalized. Perhaps the notion that the mutant races are in the majority of cases more variable than the wild type derives from the fact that those mutants were mutants of very strong effect, likely affecting dramatically the genetic underpinning of the process. For example, mutants affecting eye development severely, such as those in *ey, eya, dac, ato, L…* or *B*, as described in our paper, are associated with increased apoptosis during eye development (Halder, 1998; Bonini, 1993; Cheyette, 1994; Mardon, 1994; Jarman,

1995; Singh, 2006). We find this not surprising, as these mutants disturb retinal differentiation in one way or another and have (or must have) an impact on *dpp* expression and/or signaling. In other words, that mutants severely affecting eye development lose developmental robustness might be globally explained if they disrupt the *dpp*-mediated feedback we describe in our paper. In this sense, the explanation would not be trivial, but general instead. What happens if milder genetic perturbations are applied? When just apoptosis is blocked during the development of otherwise normal eyes, we observed two things: the eyes became slightly larger, within a range that is found in other related *Drosophila* species (for example, *D. pseudoobscura* has eyes 10% larger than those of *D. melanogaster* (Ramaekers, 2019)), but the size distribution becomes wider, also with a larger fluctuating asymmetry. Therefore, just preventing cell death, which is the process targeted by the feedback, already increases eye size imprecision (Figure 1j and 1j).

We do not know of any comprehensive study of the variability in eye size precision (i.e. FAi) in eye mutants, and we agree that such a study could give interesting insights into the mechanisms of precision control. However, in the course of an unpublished work investigating the reason why progenitor cells depend on Dpp signaling for survival, we made the following observation: the mean eye size of *dpp*-attenuated eyes (*optix>tkv-RNAi*) is highly dependent on nutrition: growing the flies in rich medium, instead of in our standard fly food, results in a milder eye reduction. However, the fluctuating asymmetry index (that is the degree of decanalization) in both conditions is comparable (see graphs below).

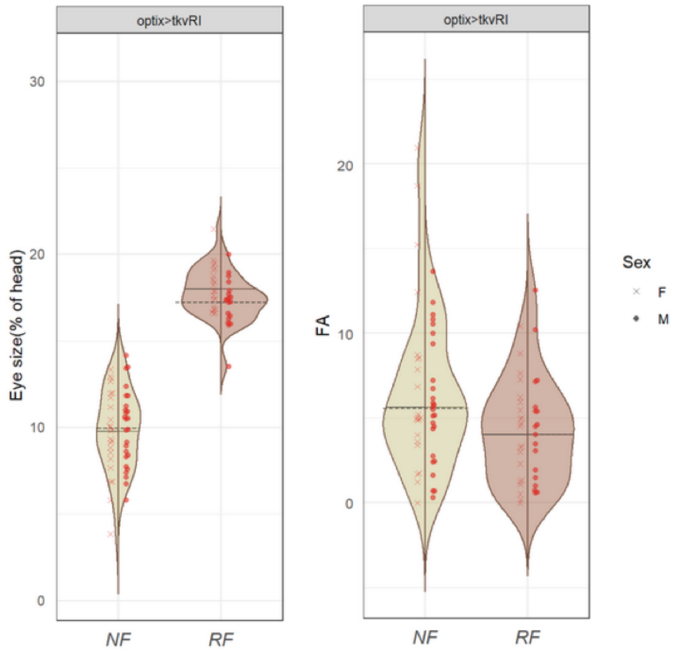

optix>tkvRNAi flies raised in normal (NF) or rich (RF) medium. Eye size (left) and FAi (right) distributions, for both males and females, are shown. The mean eye size of animals raised in NF is about 60% that of animals raised in RF and yet the FAi of eye size, which measures the degree of developmental decanalization, is very similar in both conditions.

In our opinion, these results indicate that phenotype and its “control” can be, at least, partly decoupled. But see below.

(2) Have authors produced progenitors in excess and checked whether apoptosis is increased, whether this apoptosis can be rescued by increasing Dpp signaling and

whether this has a (negative) impact on size precision? This is genetically feasible and fundamental to test their hypothesis.

R2. We have followed the reviewer's suggestion as what regards the second part of this comment ("production of progenitors in excess..."). First, we would predict that perturbations that do not affect the feedback mechanism should result in phenotypic changes (i.e. variation in eye size) *without affecting* the precision. This would be the case of progenitor proliferation: although itself subject to the feedback, it is not part of the feedback mechanism *per se*. Indeed, the new sensitivity analysis that we now include in the revised paper (under supplementary mathematical model) shows that while varying progenitor proliferation rate has a significant impact on eye size, its effect on the fluctuating asymmetry is much milder. Then, we study experimentally a genotype in which progenitors are produced in excess: This genotype is GMR>Upd, in which the expression of the cytokine and ligand of the JAK/STAT pathway Upd1 is driven in the eye by the GMR enhancer. In GMR>Upd animals, eyes grow very large (Bach 2003; Tsai and Sun 2004) (about 1,4X that of controls, Vollmer 2017). However, the FAi index of GMR>Upd and the GMR>+ controls is not significantly different. These results, which are now included in the revised version of the paper (in the main text and as a new supplementary Figure) show how a genetic perturbation causing dramatic effects on eye size does so without a loss in precision. This is explained because it does not compromise the feedback control mechanism. We sincerely thank the referee for her/his question and experimental suggestion. The new text and figure, with its legend is copied below:

"In his paper of 1942, Waddington noted that while the morphological phenotype of wild type strains was usually developmentally stable, or "canalized", "there is scarcely a mutant which is comparable in constancy with the wild type" (Waddington, 1942). That is, the phenotype of mutants is often "decanalized". We would expect decanalization of the phenotype if the mutants were affecting control feedback mechanisms. The observation that this decanalization is frequently exhibited by mutants with overt morphological alterations might indicate that most genes with major roles in the development of an organ are also part of the feedback control mechanism themselves, or affect this mechanism. In the case of eye development, these would be mutants uncoupling retinal differentiation and progenitor growth, blocking Dpp reception or its control over apoptosis in progenitors. However, perturbations that maintain the feedback mechanism intact might result in phenotypic changes, but without major effects on precision. In the eye, one such perturbation could be the variation of progenitor growth rate. This is an input to the system and subject to the feedback control, but not itself part of the feedback mechanism. Indeed the sensitivity analysis of our computational model indicates that while eye size is highly sensitive to variations in progenitor growth rate, the FAi is much less so (see Supplementary mathematical model) –that is, despite a major phenotypic change (eye size), the result of this perturbation should remain canalized. This perturbation of eye development can be induced *in vivo* by the genetic overexpression of the mitogenic cytokine and ligand of the JAK/STAT pathway *unpaired 1* (Upd) (Bach et al., 2003). Expression of Upd using the eye-specific enhancer GMR results in overgrown eyes (Bach et al., 2003), about 1,4-times those of wild type flies (Suppl. Fig. 1a,b to Figure 4). Although there is interindividual variability in rE (Suppl. Fig. 1c to Figure 4), the developmental noise of eye size, measured by its FAi, remains similar to that of control animals (Suppl. Fig. 1d to Figure 4). This latter result –the maintenance of organ size precision- is expected as long as apoptosis is operating under the feedback control. Indeed, GMR>Upd discs exhibit apoptotic signal in progenitor cells (Suppl. Fig. 1e,f to Figure 4). Therefore, the mechanism controlling eye development can accommodate changes in final organ size while maintaining its precision as variations in progenitor cell proliferation do not

interfere with the feedback control."

"Suppl. Fig. 1 to Figure 4. A mutant condition in which eye size varies dramatically preserving precision. (a, b) Heads from GMR>+ (a) and GMR>Upd (b) adult males; frontal view. While the median eye size (rE: c) of *GMR>Upd* flies is approximately 1,4 times that of control (*GMR>+*) ones (statistically significant), the fluctuating asymmetry index ("precision")(FAi: d) is indistinguishable between the two genotypes. Female (F) and male (M) distributions are shown. (e, f) Eye primordia stained for the apoptotic marker Dcp-1. Apoptotic signal is observed in both *GMR>Upd* as well as in *GMR>+* control primordia."

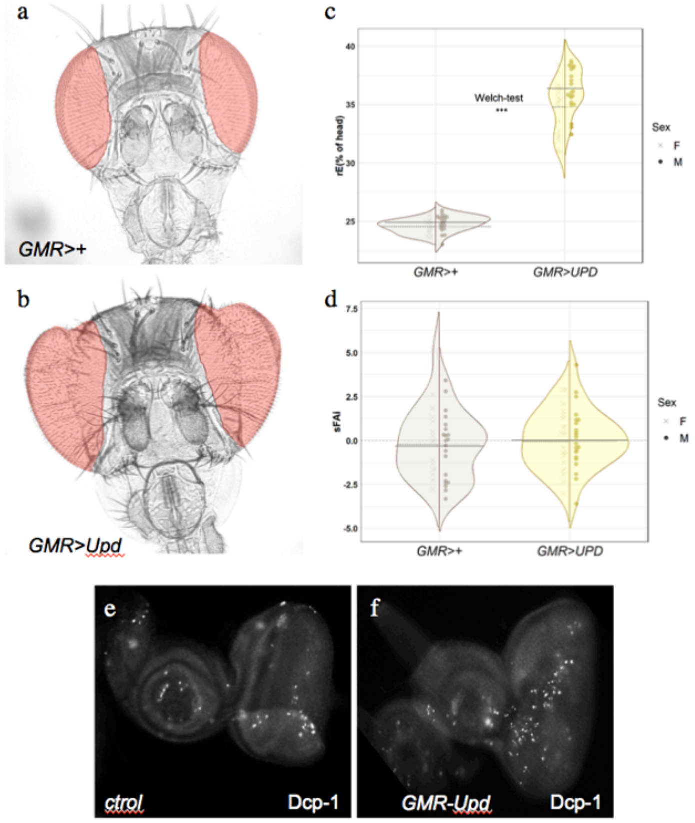

Other (essential) comments:

(1) I wonder why authors have not tried to deplete Dpp and see whether the effects on apoptosis are reproduced. This is important for the model as different BMPs and/or sources (can) exist. *Dpp h*eteroallelic combinations, in addition to reducing eye size, increase apoptosis in discs, as reported in the works by Bryant (1988) and Masucci and co-workers (1990), which were cited in our paper. We actually use these results to support our hypothesis that the Dpp signaling pathway acts as a regulator of progenitor growth through apoptosis.

(2) Apoptosis: authors should check with other tools whether cells are actually dying upon Dpp signaling inhibition (eg. TUNEL, pyknosis).

We have now used a second apoptotic marker, the cleaved, active form of caspase 3 (cas3*) to show that the attenuation of the Dpp pathway (this time attained by overexpression of the pathway's inhibitor Dad) increases dramatically cas3* signal, which supports the idea that progenitor cells deprived of this signaling enter the apoptotic pathway. To further analyze whether this is an irreversible entry into the apoptotic pathway ("whether cells are actually dying") we counterstained the DNA with DAPI. We observed that cas3* signal was associated with pycnotic nuclei, indicating

cells are indeed undergoing apoptotic death. This is now shown in a new supplementary figure (Suppl. Fig. 2 to Figure 2).

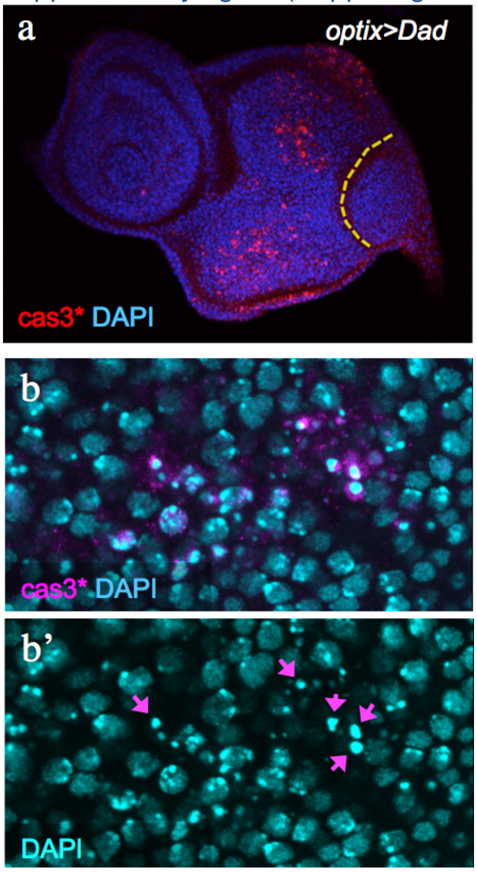

(3) Cell size should be considered in their quantifications. As a proxy, the number of ommatidia could be quantified to monitor whether the reduction in eye size correlates with a proportional reduction in ommatidia number.

We have followed the reviewer's advice and checked for ommatidial size (as a proxy of cell size) in a number of key genotypes. We do not detect significant changes in ommatidial size in these genotypes indicating that the alterations in eye size are attributable to variation in ommatidial number (which, because of the ommatidium's constant cell composition, translates into cell numbers). This is shown now in a new supplementary figure (Suppl. Fig. 2 to Figure 1).

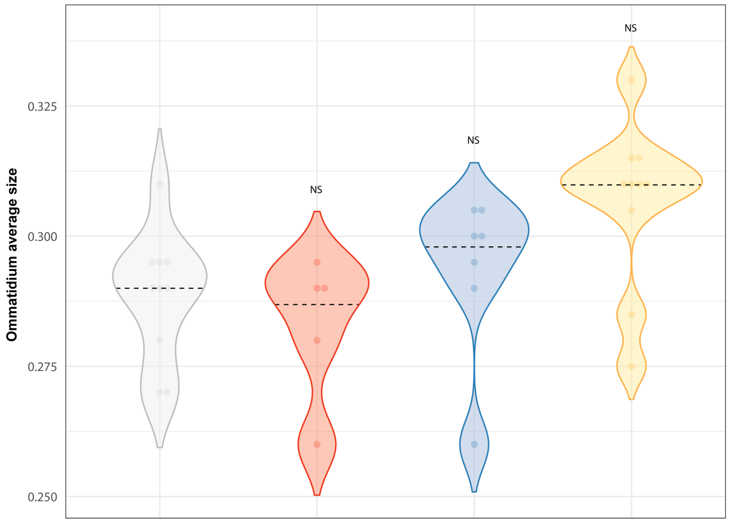

Median regression model	optix>+	optix>RHGRI	optix>tkvRI	optix>tkvRI+RHGRI
Effects	0.29	0.00	0.01	0.01
p.value	0	1	0.2187	0.5596
Predicted for group	0.29	0.29	0.30	0.31

Suppl. Fig. 2 to Figure 1. The average ommatidial size is approximately constant in different genotypes tested. The average ommatidial size was calculated as the area occupied by 10 adjacent ommatidia in the equatorial region of the eye divided by 10. Independent eyes were measured on photographs of mounted eyes of the indicated genotypes. The statistical comparison of the values obtained relative to the control *("optix>+")* did not detect significant differences among genotypes.

Minor comments:

(1) Is RHGRi a polycistronic RNAi or a synthetic miRNA?
It is a polycistronic RNAi, as indicated in the text (line 124).

(2) Apoptosis index should be defined in the text.
Although the apoptotic index is defined in the materials and methods section, we have included its description in the results section as "the apoptotic index is measured as the ratio of Dcp-1 positive pixels relative to the total pixels in the region anterior to the morphogenetic furrow of the eye primordium; see Material and Methods)".

(3) Dcp1 antibody is against the cleaved (active) form of Dcp1? Details are lacking in the text and M&M.
It is against the active, cleaved form. We have included this detail now.

(4) P values should be shown in the graphs (and not only as a supplementary table). We have included them in the figures, as the reviewer suggested.

Rev. 2:

The manuscript addresses the problem of final size control of developing tissues, particularly robustness to developmental noise, and uses the Drosophila eye imaginal disc as a model system. The question is very appropriate and much of the work is very well done.

The paper has two parts, an experimental section followed by a modeling section. The experimental section provides strong evidence that Dpp produced by cells in the optogenetic furrow inhibits the death of progenitor cells. Noting that genetic manipulations that affect this circuit lead to greater variation in eye size (both between left and right and between individuals), they postulate that Dpp's effect is part of a control circuit that serves to control eye growth and differentiation in a manner that buffers against developmental noise. It's a reasonable hypothesis, although enthusiasm should perhaps be tempered a bit by the fact that the appearance of excessive variation in response to mutations of all kinds is so common a phenomenon in biology that the now-famous term "decanalization" was coined by Waddington to describe it. Still, this reviewer has no objection to giving this hypothesis a good test, and applauds the authors for seeking to do so.

The rest of the paper describes the development and analysis of an explicit model to support the hypothesis. This is an appropriate use of modeling, and a good deal of sophisticated analysis is carried out. However, there are a number of flaws and problems with the modeling approach that likely impact the conclusions. In particular, one gets the sense that many of the assumptions made to get the model to work and enable the application of analytical tools were sufficiently ad hoc as to call the conclusions into question. Troubling assumptions include the notion that one can replace signaling over space with saturating functions of cell numbers and that developmental noise can be appropriately characterized using Langevin equations.

R: We thank the Reviewer for singling out important points in our modeling strategy. Langevin equations, as we will discuss in reply to question 4, have been widely used to model developmental noise (Ham et al. 2022), and their validity as a model for noise has been rigorously derived and discussed (Gillespie 2000). However, modeling an admittedly spatially extended system using ODEs in the way we do is, in our opinion, a valuable original contribution of this work. We will discuss this in detail below in response to question 2, and hope that after our clarifications, the Reviewer will come to appreciate the necessity, simplicity, and (in our opinion) beauty of our approach.

Specific comments on the modeling section include:

1. The diagram (Figure 2 of the supplementary section) and discussion do not match the differential equations. In the text and figure they state that "Rn cells maturate automatically into R cells." However, in their equations, the rate of Rn cell maturation into R cells is a function of R cells; in fact, it is the same function of R cells that is meant to represent Hh's effects on G cell differentiation into Rn cells. Whether this dependence was intentional or not is unclear, however given the parameter ranges explored it certainly has a large effect on the behavior of the equations, and therefore needs to be justified. In particular, there would need to be a strong justification for using the identical dependence of Rn differentiation on R as is used for the dependence of G differentiation on R. While that might seem reasonable at first, the geometry of the system ensures that the average distance of G cells from R cells is very different from the average distance of Rn cells from R cells; if a dependence on R is supposed to capture the notion that Hh only diffuses a certain distance, then one would expect to see very different dependences.
R1: The reviewer is right: the “automatically” in that sentence is misleading and has been eliminated from the text. In addition, it is true that the diagram in Figure 2 of the mathematical model section (also in Figure 3a) was oversimplified. Now, we have

amended it so that it represents the interactions modeled by the equations. Here, the concern is the use of *h(R)* in both the equations representing the time evolution of G and Rn. Its use is intentional, and the reason are two-fold: It has been shown experimentally that *both* Dpp and Hh signals act redundantly to promote the G->Rn transition (see for example Lopes and Casares 2010 and references therein). Thus, in our model, actions of Dpp and Hh in this transition have been included as depending on *a(Rn)* and *h(R)*, respectively. We have now included a sentence explaining these biological observations plus supporting references. In addition, if the width of Rn is constant (a reasonable assumption, taking into account that the experimentally determined differentiation velocity is about constant), then the rate of cells differentiating from *G->Rn* has to be related to the rate of cells exiting the Rn state (Rn->R). This results in that the equations representing the regulation of these steps share terms.

2. The authors use a non-spatial (ODE) model to represent a phenomenon that plays out in space, and therefore must incorporate a variety of simplifying assumptions that are quite ad hoc, namely three different Hill functions with arbitrary Hill coefficients and EC50 values. The desire to use ODEs to approximate what is really a PDE problem is understandable, and is occasionally justifiable, but a lot more work is required to justify this. This is because the authors seek not only to produce a model that agrees in its broad qualitative behavior with observations, they also wish to examine things like robustness and sensitivity to noise. In the end, it might be easier to at actually write out the PDEs, put in diffusion explicitly, and do a few spatial simulations, than to try to make a convincing argument that one can substitute Hill functions with numbers of cell instead.
R2: We sincerely believe that our choice of modeling strategy is appropriate for a model that is phenomenological and that makes use of limited experimental data (the time evolution of G, Rn, R…) which, in addition, does not come with spatial information (or just spatial information that is implicit in their mutual production relationships). We have used a similar approach successfully in the past for the analysis of related problems (see Vollmer et al., 2016; Lobo-Cabrera et al., 2021). It is true that, in this case, we are including non-autonomous signaling, which implies spatial relationships. However, developing a spatio-temporal model using PDEs would require including further variables and parameters (such as effective diffusion constants, characteristic lengths, etc.) for which we don't have measurements. With more parameters to estimate, the more blurred the use of the model becomes. This is not to say that generating such PDE model and the necessary data sets is not interesting -quite the contrary. It could be particularly useful to investigate not only size (which is the subject of our paper), but also shape. But let's stick to the problem we set out to analyze, eye size precision: In our model, the Hill function is used to represent a saturation event - that is equivalent to say that beyond a certain quantity, G cells are not "reachable" by the signals sent by Rn/R. This is to us, an appropriate use of a Hill function to model "distance" phenomenologically. The problem would be serious if *Kg* were a very sensitive parameter, since *Kg* represents the "lengthscale" of G where differentiation can happen. However, the parameter-sensitivity analysis we have carried out (see suggestion by this reviewer below) indicates that both rE and FAi are almost insensitive to variations in this parameter (incidentally, this parameter vas given a value of 4µm in computational experiments. If it had been given a more realistic value of 40µm (see, for example, estimates in Miguez, Garcia-Morales and Casares, 2022), the results, especially those of FAi, would have been almost identical). Therefore, once we admit a Hill function as a reasonable approximation to "distance" in our model, the exact value chosen for *Kg* is not critical. But perhaps the most reassuring result is that, with our

choice of model, we are capable of quite accurately predict the eye size and precision values of many different genotypes by using a reduced amount of experimental data. We really hope the reviewer agrees with us, even if partly, now.

3. Parameter choices are arbitrary and may be unrealistic in some cases. For example, the size of the R domain over which Hh effects reach half-saturation is set at 190μm. This reviewer is not aware whether the range of Hh spread has been measured in the eye disc, but elsewhere it tends to have a very short range, typically on the order of a few cell diameters. How would changing K_r to, say, 4 μm change the behavior of the model?

R3: It is important to keep in mind that these are effective parameters. We already had discussed in the previous version of the mathematical supplement how, in general, biological models are "sloppy" (in a technical sense), and that implies limited meaning of particular parameter values. But it is true that, specifically for the case of *Kr*, its dimensions are confusing. The term *h(R)* models the effect of Hh on the differentiation rate. We do not explicitly model Hh, under the assumption that the levels of Hh would be roughly proportional to *R* (page 1 of the Mathematical Model supplementary text). Therefore, *Kr* has dimensions of space for consistency with the dimensions of *R* (also with dimensions of space), but in fact represents the concentration of Hh for which its effect on differentiation is half saturated. Remember that we are taking *R* as a proxy for Hh levels. If we were to replace *R* by a proportional Hh concentration, the same proportionality constant would apply to *Kr* and convert it to its true dimension as a concentration. As this was not discussed in the text, this point was necessarily confusing. We thank the Referee again for calling our attention to it; in the new version we have done an effort to present these equivalences clearly. This is related to the previous discussion on the regulation of G->Rn and Rn->R: the function *h(R)* is a mean field translation of the activity of Hh in the system, with *Kr* defining the threshold for Hh activity. Different factors multiplying *h(R)* imply different effects on both regulations, but a common threshold for Hh activity. More generally, and as suggested by the reviewer, we have carried out a parameter sensitivity analysis that is presented in the revised supplemental mathematical model (novel figure 12 in that section). Furthermore, we have explored several orders of magnitude of the different noise strengths in the model, Figures 8-11.This analysis goes beyond the small perturbations of a sensitivity analysis, and shows that the apoptotic noise strength is rather constrained, Figure 8. Once this value is fixed, other noise strength parameters can vary substantially with small effects on the results, Figures 9-11. All in all, these analyses show the general robustness of the model.

4. Modeling "developmental noise" is not a straightforward process. In proliferating cell systems, the primary source of developmental noise would seem to be the fact that one has finite numbers of cells making discrete decisions probabilistically; thus the same initial conditions will lead to different outcomes when simulated multiple times. Monte Carlo simulations provide the most direct way to enumerate outcomes, but they can be a bit cumbersome to run, and difficult to analyze. Langevin noise approximations provide a faster way to explore noise effects, and enable the use of analytical tools, but they are not always appropriate for every purpose. They are often used together with chemical-type equations composed of linear terms; with proliferation equations (where species are growing exponentially) they can underestimate noise effects. In view of this it would have been a good idea to supplement the data with a few well-chosen Monte Carlo simulations.

R4: It was the fact that “Langevin noise approximations provide a faster way to explore noise effects, and enable the use of analytical tools”, which led us to choose them. Ideally, if we had the data required, we would not only include space, but also the behavior of individual cells. We agree that the best way to model noise in such a model of cellular agents would be through Monte Carlo simulations. Alas, again, we do not have the data that would constrain these simulations. Therefore, not doing it is not only a matter of convenience, but also a methodological constraint imposed by the data we have.

Regarding how appropriate Langevin noise is in our context, first we should note that Langevin dynamics can be rigorously derived from the underlying Master Equation (Gillespie 2000), and in a broad range of situations constitute excellent approximations of Monte Carlo algorithms (Gillespie 1977), simulating the full stochasticity of the Master Equation. The main microscopic limits of this approximation translate, at an observational level, in the fact that the Langevin approximation breaks down when the noise distributions are multimodal (Ham et al., 2022). In our work, the experimental results show noise distributions with a single mode (Figures 1 and 4), therefore allowing the use of Langevin dynamics to model them.

A series of criticisms on the use of Langevin dynamics for biological processes have their root in the work of McAdams and Arkin (McAdams and Arkin 1999). However, those are valid only for Langevin equations with additive noise (Bialek 2000), that are not correct approximations of the underlying stochastic dynamics (Gillespie 2000). In our case, we use the full Langevin dynamics as derived by Gillespie and others earlier. These dynamics are multiplicative: the noise strength is not constant, but depends on the trajectory of the dynamical system. In this way, exponential dynamics also imply exponential increase in noise strength, as we have illustrated for epidemic models using a full Bayesian approach (Castro et al. 2020). In our work, we have not only considered the multiplicative nature of the noise term, but used a non-diagonal diffusion matrix. Doing this, we disregarded the frequent simplification of a shared noise strength for the different processes involved in the dynamics.

We have already seen how including the correct Langevin approximation, with multiplicative noise, avoids the problem of underestimating noise effects. Furthermore, the parameters determining the noise strength of each process in our model are fits: therefore, they are chosen so that they reproduce the correct amount of noise in our observations. We now discuss these points more carefully in the revised manuscript.

5. A biologically useful control system should not only reject noise disturbances, it should also exhibit an appropriate degree of parametric robustness, i.e. it should not require that parameters—including initial conditions—be fine-tuned in order to produce a reliable output. Whereas the authors may feel that a full exploration of the robustness properties of their model is beyond the scope of this paper, it behoves them to show that the model they come up with is at least not excessively fragile.
R5: We thank the Reviewer for this suggestion. A more systematic assessment of parametric robustness indeed contributes to a clearer understanding of the weight of the different parameters and the process they represent carries in the results. For this reason, we have now performed a sensitivity analysis. This analysis is now presented in Figure 12 of the mathematical model supplement and discussed in a new section of the supplement.

Regarding initial conditions, they are perhaps the only parameters that are very well constrained by our data. As shown in Figure 3e, the initial value of G is restricted to a

narrow range by our data. So is the initial total eye size, although in this case a small trade-off between the initial values of *Rn* and *R* is possible. We now show that including some noise, within biological ranges, in the initial conditions has no effect on our conclusions. Here we see the medians of *rE*, the average intraindividual eye size, and *FAi*, the intraindividual fluctuating asymmetry, from simulations of 50 pairs of eyes with the same initial conditions:

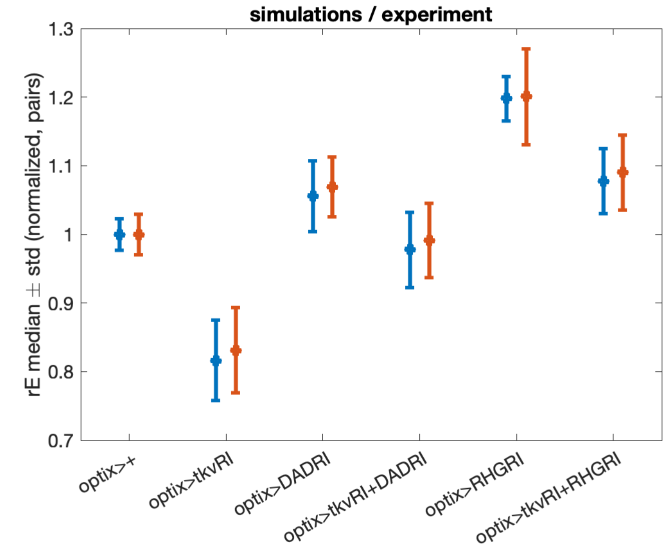

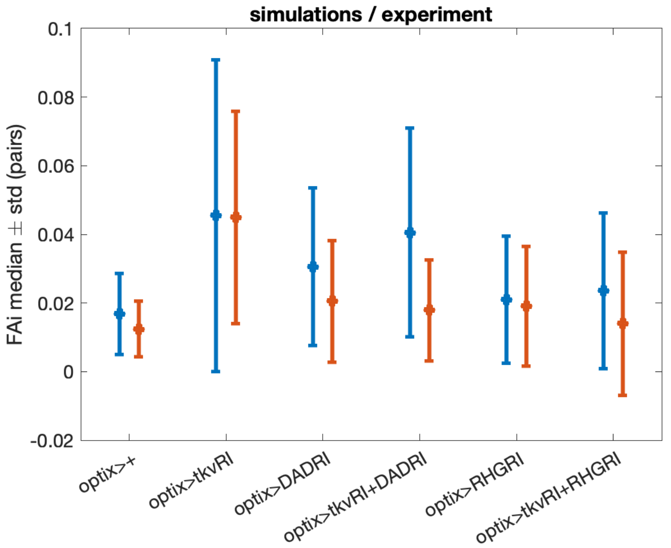

First, we proceed to add noise to the initial conditions of different flies, but keeping the pair of eyes of the same fly with the same initial conditions. Adding independent random perturbations to these initial conditions from a uniform distribution within ±10% of the initial value of each variable (if the initial value is x0, we now allow for values obtained from the interval (x0-0.1x0 , x0+0.1x0)), we see bigger variability in *rE*, but the results are still robust. In particular, *FAi* remains very similar to the case without noise:

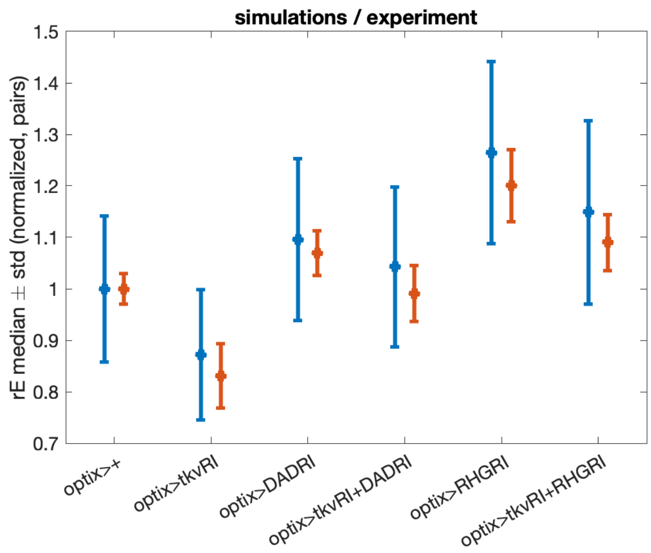

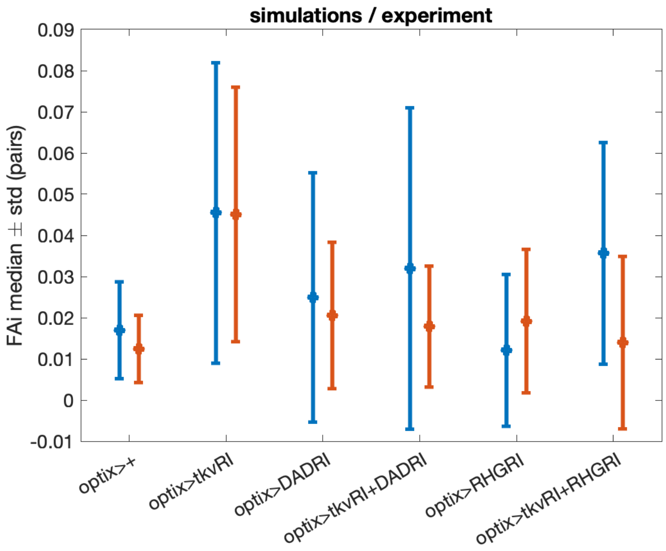

Then, we add no noise to the initial conditions of one eye of each fly, and a random perturbation to the initial conditions of the other from a uniform distribution ±1% of the value of the initial condition (if the initial value is x0, we now allow for values obtained from the interval (x0-0.01x0 , x0+0.01x0)). This is a non-negligible noise, note that the giant 10% of the previous figure was for eyes of different flies, but we would not expect the two eyes of the same fly to start developing so differently. The average eye size *rE* is robust to this intrafly noise, while small changes in *FAi* can be observed:

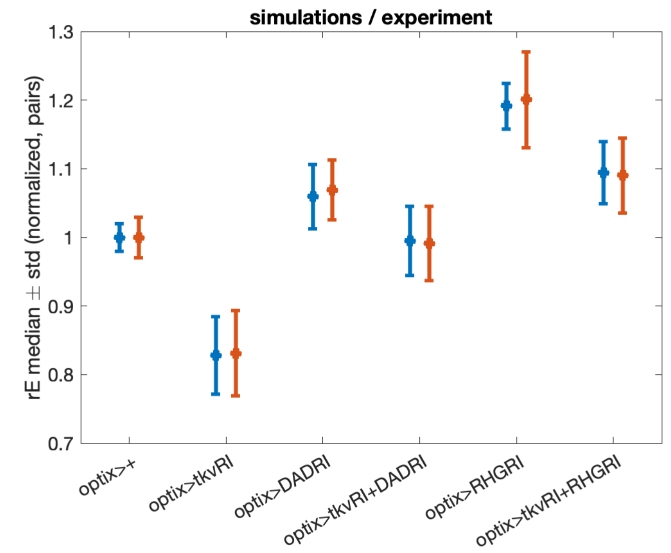

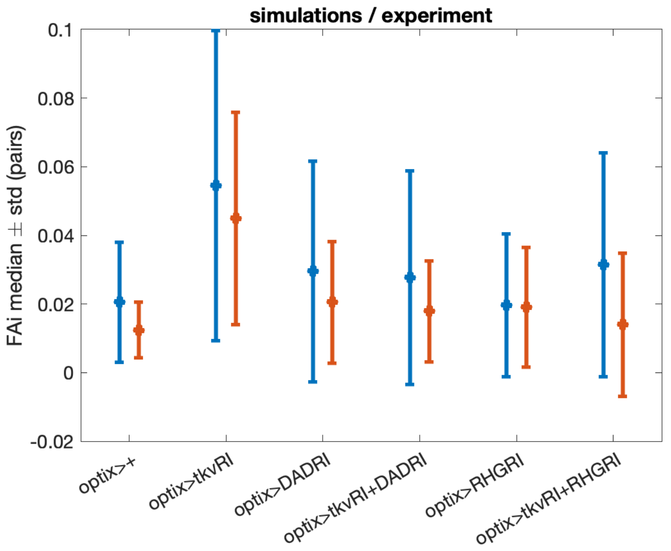

This increase in the asymmetry of optix>+ was expected, as that is exactly what we are varying in this initial conditions. The important point is to note that the process continues to be robust, and the increase in asymmetry could have been compensated by decreasing the dynamical noise in the Langevin equations of the model. Note that actually there is no systematic change of *FAi* for other conditions: the small variations are well within the uncertainty range.

Finally, combining the 10% interfly noise and 1% intrafly noise, we observe both effects simultaneously:

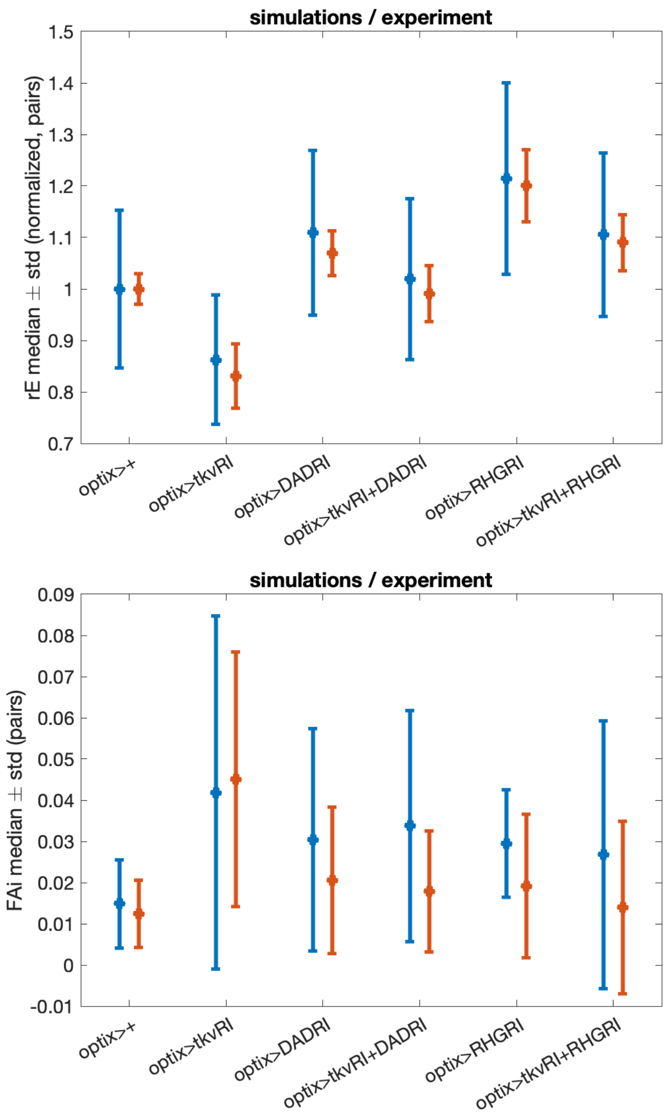

The system is totally robust to this noise in initial conditions, and the small variations in asymmetry could have been compensated by weaker dynamical noise. We had already worked with this noise in the initial conditions to convince ourselves of the robustness of the model, and decided not to include it in the manuscript to simplify the discussion. It implies adding new parameters that muddle the discussion about the sources of dynamical noise, without adding relevant information. Therefore, we now mention in the mathematical model supplement that the results are robust to noise in the initial conditions, but since it is difficult to disentangle this static noise from dynamic fluctuations, we do not present this analysis to keep the discussion simple.
